# Protease production by *Serratia liquefaciens* NRC1 using fish gut waste as a sustainable approach to antimicrobial peptide generation and combating *Candida auris* acquired resistance

**DOI:** 10.1186/s12934-025-02767-0

**Published:** 2025-07-02

**Authors:** Shaymaa A. Ismail, Heba M. Shalaby, Amira A. Hassan, Marwa Mahmoud, Bahgat Fayed

**Affiliations:** 1 Department of Chemistry of Natural and Microbial Products, Pharmaceutical and Drug Industries Research Institute, National Research Centre, 33 El Bohouth St., Dokki, P.O.12622, Giza, Egypt; 2 Human Medical Molecular Genetic Department, Human Genetics and Genome Research Institute, National Research Centre, 33 El Bohouth St., Dokki, P.O.12622, Giza, Egypt; 3 Stem Cell Research Unit, Medical Research Centre of Excellence, National Research Centre, 33 El Bohouth St., Dokki, P.O.12622, Giza, Egypt

**Keywords:** *Candida auris*, Resistance, Protease, Peptides, Fish waste, Plackett-Burman design

## Abstract

**Background:**

The global rise of antimicrobial resistance has accelerated the search for sustainable and eco-friendly therapeutic alternatives. This study evaluates fish gut waste (FGW) as a low-cost, renewable substrate for producing antifungal peptides through the proteolytic activity of *Serratia liquefaciens* NRC1.

**Result:**

Optimization of protease production using statistical designs resulted in a fourfold increase in enzyme yield. The protease demonstrated stability at neutral pH and moderate temperatures (40–50 °C), and efficiently hydrolyzed complex proteins such as collagen and keratin. Peptides generated from FGW hydrolysis exhibited significant antifungal activity against *Candida auris* (*C. auris*), with a MIC₅₀ of 5.1 ± 0.08 mg/ml. Unlike fluconazole, repeated peptide exposure did not induce resistance, nor did it alter the expression levels of key resistance genes (*CDR1*,* ERG11*), as confirmed by qRT-PCR. Peptide profiling using MALDI-TOF/MS, coupled with in silico analysis via AMPfun, identified multiple candidates with predicted antifungal properties.

**Conclusion:**

This research highlights the potential of fish gut waste-derived peptides as a sustainable and effective antifungal strategy against *C. auris*, offering an alternative to conventional antifungal drugs.

**Supplementary Information:**

The online version contains supplementary material available at 10.1186/s12934-025-02767-0.

## Background

The disposal of fish gut waste (FGW) is a significant environmental issue that affects both marine and terrestrial ecosystems. Fish gut waste is produced in large quantities by the global fishing industry. Decomposing fish waste releases harmful chemicals and pathogens into the water, harming marine biodiversity and ecosystems [1].

However, FGW can provide an excellent nutrient source for microbial growth, useful in enzyme production process, which is largely governed by the cost related to the growth media [2]. FGW consists of various organic materials, including proteins, lipids, and carbohydrates, which are essential for microbial metabolism. Additionally, FGW contains vitamins, minerals, and other micronutrients that can support microbial proliferation and the production of wide range of valuable enzymes such as proteases, lipases, and amylases [3, 4]. Chitin, a polysaccharide found in the exoskeletons of marine organisms and the cell walls of fungi, is particularly abundant in FGW and serves as an essential carbon source for microbial metabolism. The presence of chitin could also contribute to the antifungal activity of certain enzymes, as chitin components are a key target in fungal cell walls [5, 6]. Thus, this application not only addresses the disposal issues associated with fish waste but also contributes to sustainable biotechnological processes. Proteolytic enzymes can efficiently break down organic matter, including FGW, into simpler, non-polluting compounds. For instance, proteases are enzymes that break down proteins into peptides and amino acids [4]. These peptides, once released, can have various valuable applications. They may possess bioactive properties suitable for pharmaceuticals, such as antimicrobial or antioxidant capabilities [7]. Given the growing demand for antimicrobial agents and the limited availability of traditional resources, the use of renewable carbon sources, such as FGW, for AMP production is crucial for developing sustainable biotechnological processes. As conventional sources of antimicrobial agents, such as chemical synthesis or plant-based products, face challenges like limited availability, environmental impact, and high production costs, the reliance on renewable waste materials such as FGW provides a viable alternative [8]. Utilizing FGW not only reduces the environmental burden associated with waste disposal but also contributes to the circular economy by converting waste into valuable bioactive compounds. This approach can significantly reduce the pressure on traditional natural resources while offering an environmentally friendly and cost-effective pathway for the large-scale production of AMPs.

Of interest, antimicrobial peptides (AMPs) have attracted significant attention for their diverse applications in combating microbial infections across various fields [9]. These peptides exhibit potent antimicrobial activity against a broad spectrum of pathogens, including bacteria, fungi, viruses, and even parasites [9, 10]. They have several mechanism of actions involves disrupting microbial cell membranes, inhibition of protein biosynthesis, inhibition of nucleic acid biosynthesis and inhibition of cell division, making it difficult for pathogens to develop resistance compared to conventional antibiotics [11, 12].

Given these, AMPs can hold particular promise in addressing the growing concern of *Candida auris* (*C. auris*) infection. It was first identified in Japan in 2009 and has since been reported all over the contents [13]. Its emergence as a pathogen is concerning because it represents a new challenge for healthcare systems worldwide [14]. It is known to spread in healthcare settings, including hospitals and nursing homes. It can survive on surfaces and medical equipment for extended periods, leading to outbreaks that are difficult to control [15]. Infections caused by *C. auris* can be severe, particularly in patients with weakened immune systems or underlying health conditions [16]. The mortality rate for *C. auris* infections is estimated to be up to 66%, making it a highly dangerous pathogen [17]. Further, *C. auris* is challenging to identify using standard laboratory methods, often leading to misidentification and delayed treatment [18]. Accurate identification usually requires specialized laboratory techniques, such as matrix-assisted laser desorption/ionization time-of-flight (MALDI-TOF) mass spectrometry or DNA sequencing [19].

In addition to these identification challenges and severe health impacts, one of the most alarming features of *C. auris* is its resistance to multiple classes of antifungal medications [15, 20, 21]. Many isolates are resistant to at least one antifungal drug, and some are resistant to all three major classes of antifungals (azoles, echinocandins, and polyenes). This makes treating infections caused by *C. auris* particularly difficult. *C. auris* can acquire resistance to antifungal drugs, including fluconazole and caspofungin, through mutations and overexpression of key genes such as *TAC1B*,* CDR1*, and *ERG11*, which are involved in drug resistance mechanisms like efflux and target modification [21, 22].

While AMPs have been studied for their activity against various pathogens, their use against *C. auris* has not been explored before. Additionally, the use of FGW-derived protease to generate antifungal peptides active against *C. auris* has not been previously investigated.

To address the acquired resistance capability of *C. auris*, it is preferable to develop new treatments that can target multiple cellular sites or work in a non-specific manner [23]. Since AMPs can act on different sites of action, we aim in the present study to produce microbial protease enzymes that can generate AMPs using FGW as a substrate. We evaluated the effectiveness of these AMPs against *C. auris* and investigated their potential to combat the pathogen’s acquired resistance for the first time to our knowledge.

This work could lead to the development of new, effective treatments for *C. auris* infections, expanding the arsenal of tools available to healthcare providers. Further, using FGW for enzyme production not only adds value to a waste product but also enhances environmental sustainability.

## Materials and methods

### Microorganism

A proteolytic bacterial strain was isolated from fish gut waste in the department of chemistry of natural and microbial products, National research centre, Dokki, Giza, Egypt using casein-enriched medium composed of (g/L): casein, 10; K2HPO4, 1.4; KH2PO4, 0.7; NaCl, 0.5; MgSO4, 0.1, agar, 20 and adjusted at pH 7. Identification of the isolate was initially carried out by MALDI-TOF MS performed in Children’s cancer hospital, Cairo, Egypt. After that, molecular identification of the isolate was carried out in which the genomic DNA was extracted using Gene JET Genomic DNA purification kit (Thermo Scientifc, Lithuania) as instructed by the manufacturer. PCR amplification of the 16s rRNA gene was performed using the following primers: forward primer 8 F (5′-CAG GCC TAA CAC ATG CAA GTC-3′) and reverse primer 1492R (5′-GGG CGG GGT GTACAA GGC-3′). The PCR amplification conditions were 4 min of preheating at 95 °C, 30 s denaturation at 95 °C, 45 s of primer annealing at 50 °C, 1 min extension step at 72 °C, and post cycling extension of 10 min at 72 °C for 35 cycles. The reactions were carried out in a thermal cycler (Applied Biosystem Thermal Cycler, USA). The PCR product was initially analyzed using agarose gel electrophoresis then extracted from the gel using promega Wizard Genomic DNA Purification Kit. The purified PCR product was sequenced at Macrogen, Seoul, Korea. Analysis of the obtained sequence was performed using the BLAST (N) program of the National Center of Biotechnology Information (NCBI) (Rockville Pike, Bethesda MD, USA). Multiple sequence alignment was done using the Clustal W 2.1 program. The phylogenetic tree was constructed using neighbor joining method by MEGA. X [24]. Finally, the data was submitted to the GenBank and the isolated strain received an accession number.

### Protease production

#### Preparation and analysis of the FGW for enzyme production

The collected local FGW of Nile tilapia was processed according to the method described by Ramkumar et al. [25]. Briefly, the collected waste was washed under running tape water in order to remove the existent blood then boiled for 20 min, dried at 50 °C, ground and stored under freezing conditions until further use. The prepared substrate was subjected to total protein determination by kjeldahl method [26], total lipid by Folch extraction method [27], total carbohydrate by phenol sulphuric colorimetric method [28], moisture and ash content gravimetric analysis [29].

#### Submerged fermentation and protease production using FGW substrate

The prepared substrate was utilized in a submerged fermentation process conducted in 250 mL Erlenmeyer conical flasks for enzyme production. The production medium consisted of the following components (w/v %): FGW (1%), K_2_HPO_4_ (0.14%), KH_2_PO_4_ (0.07%), NaCl (0.05%), MgSO_4_ (0.01%), and was adjusted to pH 7. The medium was inoculated with 5% (v/v) of a pre-cultured nutrient broth inoculum, which had been cultivated for 1 day. Following inoculation, the culture was incubated for 2 days at 37 °C with shaking at 4 x g. After incubation, the culture was centrifuged at 4 °C and 2490 x g for 10 min, and the cell-free supernatant was collected for subsequent experiments.

#### Assessment of protease activity

Quantification of the produced enzyme was performed following the method described by Horikoshi with modifications [30]. The procedure involved preparing a reaction mixture by adding 1 mL of the suitably diluted cell-free culture to 1 mL of a 1% casein solution, prepared in 0.2 M phosphate buffer at pH 7. The mixture was incubated for 30 min at 50 °C. Following incubation, 2 mL of 15% trichloroacetic acid was added, and the mixture was centrifuged at 7155 x g for 10 min at 4 °C. The absorbance of the clear supernatant was measured at 280 nm, with the blank consisting of trichloroacetic acid precipitated reaction before thermal incubation. The proteolytic activity was quantified based on the enzyme’s ability to liberate amino acids and non-precipitated peptides equivalent to 1 µmol of tyrosine per minute. Enzyme activity was expressed as U/mL, where 1 unit (U) corresponds to the release of 1 µmol of tyrosine per minute. A standard curve was prepared using a known concentration of tyrosine to ensure accurate quantification of protease activity.

### Optimization of protease production

#### Single-variable-at-a-time

The impact of different nutritional and cultural conditions including substrate concentration (0.2-4%), pH [5–10], temperature (25–44 °C) and incubation period (1–8 days) on the enzyme productivity were evaluated in which each experiment was carried out under the conducted optimized conditions.

#### Statistical optimization

To optimize the nutritional and cultural conditions of the fermentation process, two sequential approaches were employed: initially, the Plackett-Burman design was utilized to identify key influencing variables, followed by the application of the Central Composite Design to fine-tune and optimize these variables [31, 32].

##### Plackett-Burman design

Seven independent variables (FGW, NaCl, KH_2_PO_4_, K_2_HPO_4_ and MgSO_4_ concentrations (%) in addition to pH and inoculum size percentage) were evaluated at -1 and + 1 as shown in Table 1. The effect of each variable was determined using the formula:

1 $$ {\rm{E}}\left( {{\rm{Xi}}} \right)\,{\rm{ = }}\,{\rm{2}}\left( {{\rm{\Sigma }}\,{\rm{Mi + }}\, - {\rm{Mi}} - } \right){\rm{/N}} $$

where E(Xi) is the variable effect, Mi − and Mi + represent the protease activity at the lower (-1) and higher (+ 1) levels of the variable, respectively, and N is the number of trials [33].

**Table 1:** Plackett-Burman design showing the examined variables and their actual examined values

Variable		Level	
Name	Unit	Low (-1)	High (+ 1)
FGW concentration	%	1	2
NaCl concentration	%	0.05	0.1
KH_2_PO_4_ concentration	%	0.07	0.28
K_2_HPO_4_ concentration	%	0.14	0.56
MgSO_4_ concentration	%	0.01	0.04
pH	-	6	7
Inoculum size	%	5	10

##### Central composite design

The variables with the most significant effects were further optimized using Central Composite Design. This approach involved studying three variables at two levels across 19 experiments, which included a full factorial design with 8 experiments, 6 axial points, and 5 replicates of the central points. The actual examined values of each variable are illustrated in Table 2. The relationship between the variables and protease activity was analyzed based on the following equation:

2 $$ {\rm{Y = }}\,{\rm{B0}}\,{\rm{ + \Sigma }}\,{{\rm{B}}_{\rm{i}}}{{\rm{X}}_{\rm{i}}}{\rm{ + \Sigma }}\,{{\rm{B}}_{{\rm{ij}}}}{{\rm{X}}_{\rm{i}}}{{\rm{X}}_{\rm{j}}}{\rm{ + \Sigma }}\,{{\rm{B}}_{{\rm{ii}}}}{{\rm{X}}_{\rm{i}}}^{\rm{2}} $$

where, Y: is the predicted protease activity; B0, B_i_, Bij and Bii are the intercept of the model, linear, cross product and quadratic coefficients, respectively where Xi and Xj are the coded levels of the variables under investigation [34].

**Table 2:** Central composite design showing the examined variables and their examined actual values

Variable			Level				
Name	Symbol	Unit	-2	-1	Central (0)	+ 1	+ 2
KH_2_PO_4_ Concentration	X_1_	%	0	0.035	0.07	0.105	0.14
MgSO_4_ Concentration	X_2_	%	0	0.005	0.01	0.015	0.02
pH	X_3_	-	5	5.5	6	6.5	7

### Protease precipitation and characterization

Ethanol precipitation of the enzyme produced under optimized conditions was investigated using cold ethanol at concentrations ranging from 30 to 90%. Enzyme activity for each fraction was measured as described previously, while protein content was assessed using the method outlined by Lowry et al. [35]. The specific activity of each fraction was then calculated, and the fraction with the highest specific activity was selected for further experimentation.

The proteolytic activity of the precipitated fraction was assessed across a pH range from 6 to 10.5 using 0.2 M buffers: phosphate buffer for pH 6–7, Tris/HCl buffer for pH 9, and carbonate buffer for pH 10-10.5. Both the enzyme and substrate solutions were prepared in these buffers. Additionally, enzyme stability at the optimum pH was evaluated by measuring residual proteolytic activity after pre-incubation at this pH for various time intervals up to 120 min, with the initial enzymatic activity (before pre-incubation) defined as 100%.

The effect of temperature on the enzyme’s proteolytic activity was first evaluated across a range of temperatures (30–55 °C). Subsequently, the activation energy (Ea) for the enzyme activity was calculated from the slope of the Arrhenius plot as follow:

3 $$ {\text{-Ea/R}}\,{\rm{ = }}\,{\rm{Slope}} $$

Additionally, thermal stability was assessed by incubating the enzyme at temperatures ranging from 40 to 45 °C and measuring residual enzymatic activity under optimal conditions after pre-incubation (without substrate) for various time intervals (0–120 min). The initial enzymatic activity was considered as 100%. Following this, the enzyme’s thermal stability kinetic parameters were determined as follows:

4 $$ {\text{-Ed/R}}\,{\rm{ = }}\,{\rm{Slope}}\,{\rm{of}}\,{\rm{Arrhenius}}\,{\rm{plot}}\,\left( {{\rm{ln}}{{\rm{K}}_{\rm{d}}}\,{\rm{versus}}\,{\rm{1/T}}} \right) $$

5 $$ {\rm{ln}}\left( {\rm{2}} \right){\rm{/}}{{\rm{K}}_{\rm{d}}}\,{\rm{ = }}\,{{\rm{T}}_{{\rm{1/2}}}} $$

6 $$ {\rm{ln}}\left( {{\rm{10}}} \right){\rm{/}}\,{{\rm{K}}_{\rm{d}}}\,{\rm{ = }}\,{\text{D-value}} $$

where Ed was the decay activation energy (KJmol-1), K_d_ was the thermal-deactivation rate constant, R was the gas constant (8.3145 Jmol–1 K-1), T was the temperature (K) and D-value was the decimal reduction time.

The efficiency of the produced enzyme fraction in the hydrolysis of various protein substrates (albumin, collagen, gelatin and keratin) at 1% concentration was evaluated in compare to casein as previously described.

### Production of soluble peptides from FGW

#### Proteolytic enzymatic hydrolysis

The effectiveness of the produced protease in hydrolyzing the FGW substrate was evaluated over various hydrolysis periods up to 24 h, using an enzyme-to-substrate ratio of 1.4 U/mg. The substrate was initially suspended in 0.2 M phosphate buffer at pH 7, then an equivalent volume of the enzyme solution was added, and the mixture was incubated at 40 °C. At the end of the hydrolysis period, the reaction was centrifuged at 1790 x g for 10 min at 4 °C. The protein content released into the supernatant was quantified using the method described by Lowry et al. [35], and the hydrolysis percentage was calculated as follows:

7 $$ \begin{array}{l}{\rm{Hydrolysis}}\,{\rm{percentage}}\,\left( {\rm{\% }} \right)\,{\rm{ = [Amount}}\,{\rm{of}}\,{\rm{soluble}}\,\\{\rm{protein}}\,{\rm{in}}\,{\rm{the}}\,{\rm{supernatant}}\,\left( {{\rm{mg/mL}}} \right){\rm{/Amount}}\,{\rm{of}}\,\\{\rm{the}}\,{\rm{added}}\,{\rm{FGW}}\,\left( {{\rm{mg/mL}}} \right){\rm{]}}\,{\rm{X}}\,{\rm{100}}\end{array} $$

For the isolation of soluble peptides, the clear supernatant was boiled for 10 min to denature the existed protein molecules that removed by centrifugation at 1790 x g for 10 min at 4 °C followed by air-drying of the clear supernatant. The prepared sample was stored under freezing conditions until further use.

#### Micro-structural analysis of FGW

The remaining FGW residue was examined under scanning electron microscope (Quanta 250, HRFEG, Czech) and the elemental constituents were inspected via Energy dispersive X-ray analysis (Octane pro, AMETEK^®^ EDAX, USA) in comparison with the sample before hydrolysis.

#### Analysis of the produced soluble peptides by HPLC

The amino acid composition of the dried sample was determined using the Waters PICO-TAG HPLC method [36]. This method involves three main steps: (i) sample hydrolysis in which the sample was weighed into a 25 × 150 mm hydrolysis tube, mixed with 6 N HCl, and placed in an oven at 110 °C for 24 h. After cooling, the contents of the tube were quantitatively transferred to a volumetric flask and diluted with HPLC-grade water. The diluted hydrolysate was then filtered through a 0.45 μm filter, (ii) pre-column derivatization with Phenylisothiocyanate and (iii) analysis using reverse phase HPLC. Chromatographic separation was performed on a Pico-Tag column (150 × 3.9 mm) with a 600E Multisolvent Delivery System, using a gradient of Pico-Tag solvents A and B (Waters Eluent A & B) at 38 °C and a flow rate of 1 ml/min. The Phenylisothiocyanate derivatives were detected by ultraviolet absorption at 254 nm using a 2489 UV/Vis Detector. Amino acids in the samples were identified by comparing their retention times with those of standard amino acids.

#### Proteomic analysis of the soluble peptides by MALDI TOF mass spectrometry

Proteome analysis was performed according to previously described methods with minor modifications [37, 38]. Samples were initially prepared by adding 150 µl of 8 M Urea (500 mM Tris, pH 8.5). The samples were then homogenized using an ultrasonic homogenizer, vigorously shaken, and centrifuged at 12,300 x g for 30 min at 4ºC. Chilled acetone was added to each sample, followed by incubation at -80 °C for 30 min and then at -20 °C overnight. The samples were then centrifuged again at 12,300 x g for 30 min. Peptides were digested by adding 200 mM DTT for 45 min at room temperature, followed by the addition of 1 M IAA and incubation in the dark at room temperature for 45 min. For trypsinization, trypsin containing porcine enzyme was added and the mixture was incubated overnight at 37 °C with shaking at 100 x g. Subsequently, 100% formic acid was added to acidify the sample to pH 2–3, and the samples were centrifuged for 30 min at room temperature. MonoSpin Reversed Phase Columns were used for sample trapping according to the manufacturer’s instructions. The samples were then analyzed using a NanoLC system consisting of an Eksigent nanoLC 400 autosampler attached to an Ekspert nanoLC425 pump, employing a 3 μm ChromXP C18CL, 120Å, 150 × 0.3 mm column with a flow rate of 5 µl/min and a gradient mobile phase consisting of LC-MS water containing 0.1% FA and acetonitrile containing 0.1% FA. Mass spectrometry was performed using a Sciex TripleTOF™ 5600 + with a TOF mass range of 400–1250 m/z. Data were processed using Analyst TF 1.7.1 for data acquisition, and the raw MS files from the TripleTOF™ 5600 + were analyzed using ProteinPilot (version 5.0.1.0, 4895) with the Paragon Algorithm (version 5.0.1.0, 4874). The database used was Swiss-Prot and TrEMBL, containing 75,978 proteins. The identification was considered reliable if the ProteinPilot confidence score for a peptide was ≥ 1.3 (which corresponds to a 95% confidence level for peptide identification).

The antifungal activity of the identified peptides was predicted using online algorithms for the analysis of antimicrobial activity AMPfun predictive tool as described by Chung et al. [39]. The threshold ≥ 0.6 was selected for positive decision regarding putative antifungal activity.

### Assessment the antifungal activity of the produced peptides against *C. auris*

The antifungal activity was determined using the minimum inhibitory concentration (MIC) assay, based on modified Clinical and Laboratory Standards Institute (CLSI) protocols [16, 40]. *C. auris* derived from the strain CDC_B11220 was cultivated overnight at 37 °C in Sabouraud Dextrose Broth (SD). On the following day, a 10^4^ CFU/ml suspension of this culture was incubated in a 96-well flat-bottom plate with different concentration of either fluconazole (20, 10, 5, 2.5, 1.25, 0.625 µg/ml) or microbial peptides (20, 10, 5, 2.5, 1.25, 0.625 mg/ml). Controls contains free media or *C. auris* cultures were included. The plate was incubated at 37 °C for 48 h without shaking. After incubation, *Candida* growth was assessed by measuring turbidity at OD_600_ with a microplate reader. The MIC_50_ value was identified as the lowest concentration that achieved a 50% reduction in *Candida* growth compared to the control. The assay was conducted in triplicate, and the MIC_50_ values were averaged. The MIC_50_ was calculated and graphed using GraphPad Prism (8.02, GraphPad Inc., La Jolla, CA, USA).

### Analysis of *C. auris* acquired resistance to the produced peptides in comparison with fluconazole

#### Antifungal susceptibility

*C. auris* cells (1 × 10^6^cells/ml) were incubated in SD broth for 48 h at 37 °C with either 2.5 mg/ml peptides or 1.5 µg/ml fluconazole. After incubation, the cell density was adjusted to 0.25 OD600, and the cells were transferred to fresh SD broth containing the same concentrations of the compounds and incubated under the same conditions. This process was repeated five times. *C. auris* strains grown under identical conditions but without any antifungal compounds were served as the control. Following the final incubation, the cells were collected by centrifugation, and the MIC_50_ was determined as previously described.

#### Quantitative real-time PCR (qRT‒PCR)

The expression levels of the *CDR1* and *ERG11* genes in *C. auris* cells exposed to either the produced peptides or fluconazole five times were quantified as follows: Cells were initially beaded with glass beads for 10 min then centrifuged at 12300 x g for 20 min at 4 ^o^C. Total RNA was isolated from the obtained supernatant using a total RNA purification kit (NORGEN, Auburn, WA) according to the manufacturer’s instructions. RNA quantity and quality were assessed with a NanoDrop 2000 spectrophotometer (Thermo Fisher Scientific, USA). cDNA was synthesized from 1 µg of extracted RNA using the RevertAid First Strand cDNA Synthesis Kit (Thermo Fisher Scientific, Lithuania). Quantitative real-time PCR was conducted with the Hera Plus SYBR Green qPCR Kit (Willow Fort, Birmingham, UK) following the manufacturer’s protocol to measure the mRNA expression levels of the target genes. PCR plates were analyzed using the ABI 7500 Fast System (Applied Biosystems). Melting curve analysis was performed to confirm the specificity of the amplification products. Gene expression fold changes were calculated using the comparative threshold cycle method (2^−ΔΔCt^) for relative quantification, normalized to the endogenous control β-actin (ACTB) gene [41]. The gene expression analysis data were analyzed and graphed using an unpaired t test with Welch correction. *p* < 0.05 was considered significant. The primers used were: *ACT* Forward (5’-3’): CGCTGGTTTCTCGTTACCAC and Reverse (5’-3’): CAGCAGTGGTAGAGAAGGTGT; *ERG11* Forward (5’-3’): CAAGTCGTTGATGGGTGATG and Reverse (5’-3’): GAACGATGTCACCGGTCTTT; *CDR1* Forward (5’-3’): GCCAGGTTTCTGGATTTTCA and Reverse (5’-3’): GGCCACAAGTTTGACCACTT.

### Antioxidant activity

The antioxidant activity of the produced soluble peptides was evaluated according to Brand-Williams method that based on the scavenging of DPPH [42]. A reaction mixture (4 mL) containing 100 µL of 10% w/v sample solution mixed with methanolic solution of DPPH radical (1.1 × 10^− 4^ mol/L) was observed for a decrease in absorbance at 515 nm after standing in the dark for 30 min. The antioxidant activity was expressed in mmole Trolox Equivalents (TE)/g of the dry sample.

### Anti-inflammatory activity

The RAW 264.7 macrophage cell line was obtained from the American Type Culture Collection (ATCC). The cells were cultured in RPMI-1640 medium (Roswell Park Memorial Institute) supplemented with 1% penicillin/streptomycin and 10% heat-inactivated fetal bovine serum. They were incubated in a humidified incubator with 5% CO_2_ at 37 °C and subcultured twice before the experiment. RAW 264.7 cells were suspended in RPMI medium and seeded at a density of 1 × 10^5^ cells per well in 96-well plates. After a 24-hour incubation period, the cells were treated with the sample at concentrations of 100, 50, 25, and 12.5 µg/mL and incubated for an additional hour. Subsequently, the cells were stimulated with 10 µg/mL of LPS for 24 h.

The supernatant was gently transferred to new 96-well plates for nitric oxide (NO) determination.

The NO production was assayed by measuring nitrite in the supernatants of cultured RAW 264.7 cells. The assay was carried out as described previously with slight modification [43]. After pre-incubation of RAW 264.7 cells (1 × 10^5^cells/mL) with LPS (10 µg/mL) for 24 h, the amount of nitrite, a stable metabolite of NO use as an indicator of NO production, in the culture medium was measured using the Griess reagent (1% sulfanilamide and 0.1% naphthylethylenediamine dihydrochloride in 2.5% phosphoric acid). A volume of 50 µL of the cell culture medium was mixed with 50 µL of the Griess reagent. Subsequently, the mixture was incubated at room temperature for15 min and the absorbance was measured at 540 nm in a micro plate reader. Fresh culture medium was used as a blank in every experiment.

### In-vitro wound healing assay

The migration rates of BJ-1 cells were assessed using the scratch assay method [44]. Cells were seeded at a density of 2 × 10^5^ cells per well in a 24-well plate and incubated with complete medium at 37 °C and 5% CO_2_. After 24 h, the confluent cell monolayer was horizontally scraped with a sterile P200 pipette tip. The debris was removed by washing with PBS. The cells were then treated with samples at a concentration of 50 µg/mL, while untreated cells served as a negative control. The scratch representing the wound was photographed at 0 h using phase contrast microscopy at ×40 magnification, before incubation with the samples. After 24 h of incubation, a second set of images was taken. To determine the migration rate, the images were analyzed using Image J software, and the percentage of the closed area was measured and compared with the value obtained at 0 h. An increase in the percentage of the closed area indicated cell migration.

8 $$ \begin{array}{l}{\rm{Wound}}\,{\rm{closure}}\,\left( {\rm{\% }} \right){\rm{ = }}\\\frac{{\left( {{\rm{Measurement}}\,{\rm{at}}\,{\rm{0}}\,{\rm{h}}\,{\rm{ - }}\,{\rm{Measurement}}\,{\rm{at}}\,{\rm{24}}\,{\rm{h}}} \right)}}{{{\rm{Measurement}}\,{\rm{at}}\,{\rm{0}}\,{\rm{h}}}}{\rm{ \times 100}}\end{array} $$

### Statistical analysis

The MIC_50_ was calculated and graphed using GraphPad Prism (8.02, GraphPad Inc., La Jolla, CA, USA). The gene expression analysis data were analyzed and graphed using an unpaired t test with Welch correction. *p* < 0.05 was considered significant. The results were given as the average ± standard deviation for triplicate experimental runs with duplicate measurements of each replicate. Excel data analysis was used for regression analysis, and the Central Composite Design was analyzed by Design-Expert 12 statistical software.

## Results

### The isolated strain was identified as *Serratia liquefaciens* NRC1

**Fig. 1:**
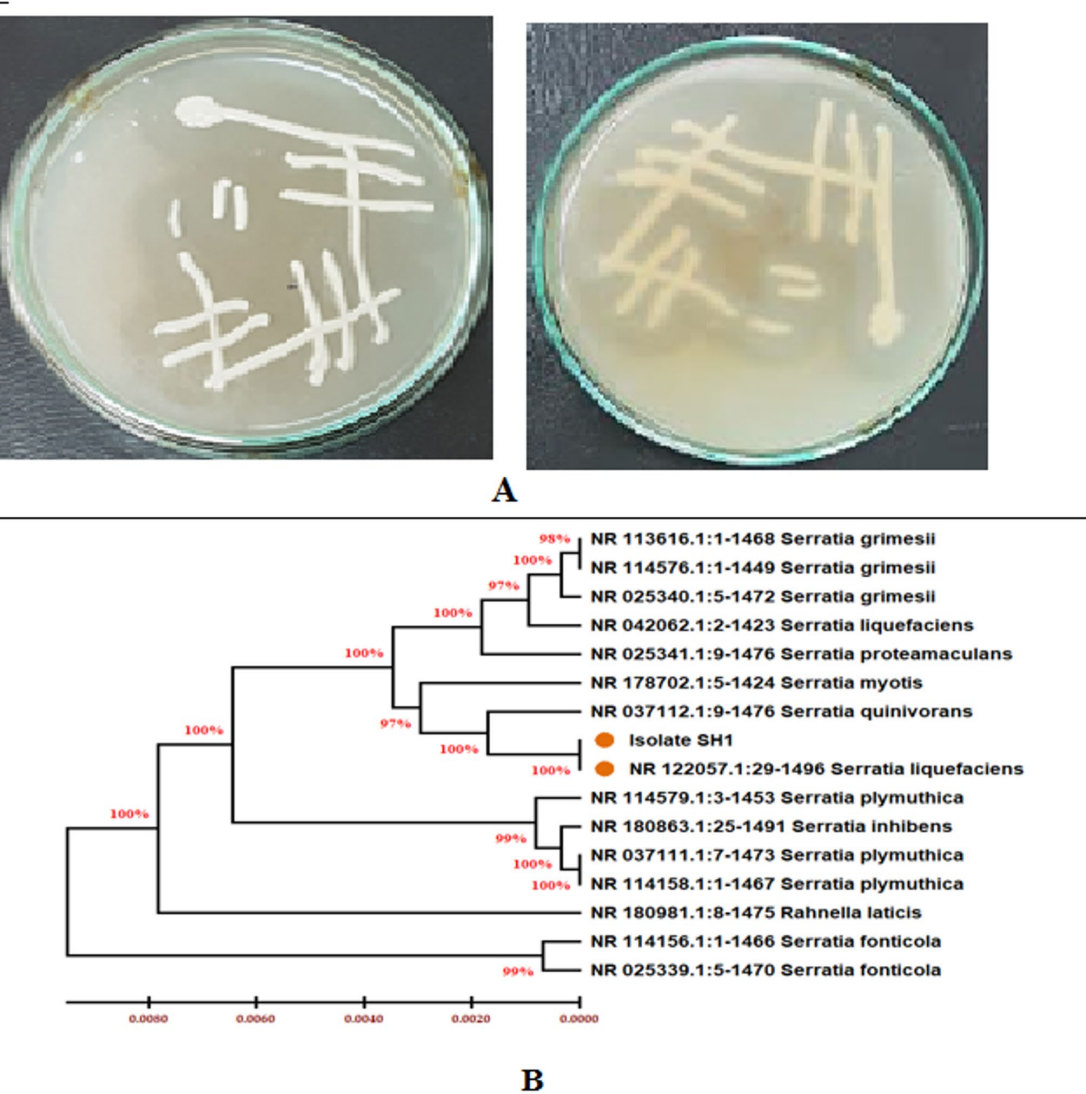
Identification of the isolated strain, (**A**) Hydrolytic activity of casein-enriched medium, (**B**) The phylogenetic tree of the isolated strain

The isolated strain used in the current study was preliminary selected on the base of its ability for the hydrolysis of casein and production of hollow zone in agar plate as shown in Fig. (1a). The isolate was a Gram-negative rod-shaped strain that belong to the genus *Serratia* as indicated by matrix-assisted lased desorption ionization-time-of-flight mass spectroscopy analysis (MALDI-TOF/MS).

Molecular identification of the isolate was carried out in which the PCR product of the 16s rRNA gene was sequenced. BLAST result indicated 98.84% identity percentage of the isolated strain with *Serratia liquefaciens*. The obtained sequence was submitted to the GenBank under the name *Serratia liquefaciens* NRC1 and received accession number of PP038128. Moreover, the constructed phylogenetic tree was shown in Fig. (1b).

### *Serratia liquefaciens* NRC1 produced protease enzyme using FGW as a substrate

In the current study, FGW was used as a substrate for inducing the production of protease using the isolated strain. Analysis of the used substrate indicated that it was rich in protein (50%) followed by lipid (16.3%) and carbohydrate (0.7%). In addition, the moisture and the ash content were estimated as 15.3 and 17.7%, respectively. Herein, we initially adapted the single-variable-at-a-time method for optimizing the production of proteolytic enzyme using the isolated strain. On the base of the obtained results illustrated in Table 3, the highest *Serratia liquefaciens* NRC1 protease activity (3.429 U/mL) was obtained via the fermentation of 1% of FGW in a production medium adjusted at pH 7 with cultured period of 2 days at 30 °C.

**Table 3:** Protease enzyme production following the single-variable-at-a-time method

Incubation period (Days)	1	2	3	6	8
Enzyme activity (U/mL)	0.396 ± 0.012	1.254 ± 0.035	0.245 ± 0.000	0.085 ± 0.006	0.068 ± 0.003
Substrate concentration (% w/v)	0.2	0.5	1	2	4
Enzyme activity (U/mL)	0.087 ± 0.004	0.134 ± 0.011	1.356 ± 0.013	0.736 ± 0.013	0.252 ± 0.003
Temperature (°C)	25	30	37	44	
Enzyme activity (U/mL)	1.098 ± 0.006	3.299 ± 0.173	1.317 ± 0.003	0.693 ± 0.013	
pH	5	7	8	9	10
Enzyme activity (U/mL)	2.679 ± 0.204	3.429 ± 0.164	2.620 ± 0.028	1.950 ± 0.089	1.321 ± 0.179

### Production of protease enzyme was optimized by adapting statistical optimization

The influence of seven variables on the enzyme productivity was examined by applying Plackett-Burman design in which each variable was examined at -1 and + 1 levels as shown in Table 4. The production of protease reached 4.69 U/mL following Plackett-Burman design. The regression analysis (Table 5) of the obtained data indicated that the coefficient of determination (R²) value was 0.96, which means that 96% of the variation in the response variable can be explained by the model. This high R² value reflects a strong fit and suggests that the model has excellent predictive accuracy. The main effects of the examined variables were calculated and the results indicated that the pH, MgSO_4_ and KH_2_PO_4_ concentrations possessed negative effects while NaCl and K_2_HPO_4_ concentrations possessed positive effects. The negative effect estimated that the variable higher effect was achieved at its lower level and the positive effect was vice versa. Therefore, each variable was adjusted in the second step of optimization at the value possessed higher effect. Moreover, the insignificant variables (the concentration of FGW and the inoculum size) were adjusted at their negative values.

**Fig. 2:**
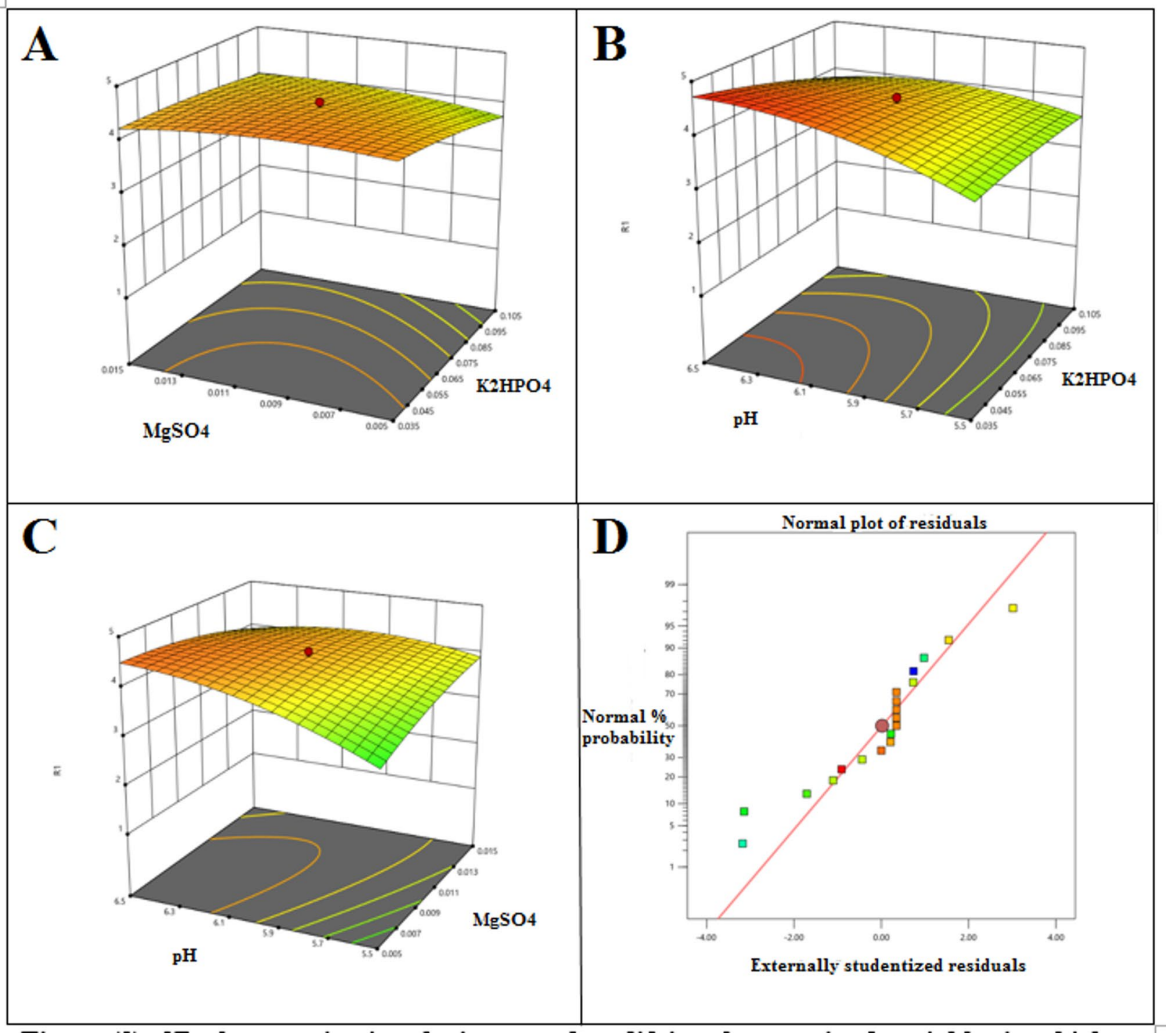
The impact of modifying the examined variables in which the obtained protease activity was the response (R1). (**A**-**C**) Each plot monitored two variables and kept the third fixed at its central level, (**D**) The residuals normal plot

The highest significant three variables were optimized by applying a central composite design as shown in Table 6. The obtained data (Table 7) fitted a quadratic model in which the ANOVA indicated the overall significance of the applied design since the F value was 11.72 with *p*-value of 0.0006. The regression analysis indicated that the R^2^ value for the applied design was 0.9214, indicating its accuracy in addition to its efficiency in explaining 92.14% of the variability in the response. Moreover, the analysis indicated the significance of the linear (X_1_, X_3_) and the quadratic (X_3_^2^) model terms in addition to the interactive term between MgSO_4_ concentration and the pH (X_1_ × _3_) that was clearly estimated by the steeply curves as shown in Fig. 2 (a-c). The second-order equation for the predication of the enzyme activity was.

9 $$ \begin{array}{l}{\rm{Y = }}\,{\rm{ - 49}}{\rm{.544}}\,{\rm{ + }}\,{\rm{75}}{\rm{.99261}}{{\rm{X}}_{\rm{1}}}{\rm{ + }}\,{\rm{832}}{\rm{.80913}}{{\rm{X}}_{\rm{2}}}{\rm{ + }}\,{\rm{15}}{\rm{.05849}}{{\rm{X}}_{\rm{3}}}\\{\rm{ + }}\,{\rm{562}}{\rm{.02215}}{{\rm{X}}_{\rm{1}}}{{\rm{X}}_{\rm{2}}}{\rm{ - }}\,{\rm{13}}{\rm{.50719}}{{\rm{X}}_{\rm{1}}}{{\rm{X}}_{\rm{3}}}{\rm{ - 126}}{\rm{.98385}}{{\rm{X}}_{\rm{2}}}{{\rm{X}}_{\rm{3}}}\\{\rm{ - }}\,{\rm{46}}{\rm{.50611}}{{\rm{X}}_{\rm{1}}}^{\rm{2}}{\rm{ - }}\,{\rm{5129}}{\rm{.38173}}{{\rm{X}}_{\rm{2}}}^{\rm{2}}{\rm{ - 1}}{\rm{.01539}}{{\rm{X}}_{\rm{3}}}^{\rm{2}}\end{array} $$

where, Y expressed the predicted protease activity and X_1_, X_2_, and X_3_ were the concentration of KH_2_PO_4_, concentration of MgSO_4_ and the initial pH, respectively.

The absolute average deviation (AAD) was calculated according to Eq. (9) and it was 12.83.

10 $${\rm{AAD}} = \{ [\sum {_{{\rm{i}} = 1}^{\rm{p}}} (\left| {{\rm{Residual}}} \right|/{\rm{Yexp}})]/{\rm{P}}\} {\rm{v}}\:{\rm{X}}\:100$$

where Yexp was the observed protease activity, P was the number of experiments and the residuals were the subtracting values of the observed and the predicted results of the enzyme activity. In addition, by plotting the residuals normal plot (Fig. 2d), the plot estimated that the residuals were normally distributed. Validation of the model was carried out by applying the predicted optimized conditions (0.04% concentration of KH_2_PO4, 0.007% concentration of MgSO_4_ and initial pH of 6.42 that lead to the production of 5.073 U/mL that was close to the predicted optimal activity (4.806 U/mL).

**Table 4:** Plackett-Burman design

Run number	FGW concentration (%)	NaCl concentration (%)	KH_2_PO_4_ concentration (%)	K_2_HPO_4_ concentration (%)	MgSO_4_ concentration (%)	pH	Inoculum size (%)	Protease activity (U/mL)
1	− (1)	− (0.05)	− (0.07)	+(0.56)	+(0.04)	+(7)	− (5)	2.25
2	+(2)	− (0.05)	− (0.07)	− (0.14)	− (0.01)	+(7)	+(10)	2.75
3	− (1)	+(0.1)	− (0.07)	− (0.14)	+(0.04)	− (6)	+(10)	2.96
4	+(2)	+(0.1)	− (0.07)	+(0.56)	− (0.01)	− (6)	− (5)	4.69
5	− (1)	− (0.05)	+(0.28)	+(0.56)	− (0.01)	− (6)	+(10)	3.31
6	+(2)	− (0.05)	+(0.28)	− (0.14)	+(0.04)	− (6)	− (5)	2.20
7	− (1)	+(0.1)	+(0.28)	− (0.14)	− (0.01)	+(7)	− (5)	2.53
8	+(2)	+(0.1)	+(0.28)	+(0.56)	+(0.04)	+(7)	+(10)	2.26

**Table 5:** Multiple regression analysis

Examined variable	Protease activity analysis			
	Coefficient	Standard error	t-statistics	*P*-value
Intercept	8.257094			
FGW concentration (%)	0.213311	0.106136	2.009793	0.079304
NaCl concentration (%)	9.707988	2.122714	4.573384	0.001818
KH_2_PO_4_ concentration (%)	-2.78449	0.505408	-5.50938	0.000567
K_2_HPO_4_ concentration (%)	1.233899	0.252704	4.882783	0.00122
MgSO_4_ concentration (%)	-30.0849	3.537857	-8.5037	2.81E-05
pH	-0.84239	0.106136	-7.93691	4.62E-05
Inoculum size (%)	-0.0204	0.021227	-0.96113	0.364632
**Summary of the model**				
Multiple R	0.98193			
R^2^	0.964187			
Adjusted R^2^	0.93285			
Standard Error	0.212271			

**Table 6:** Central composite design

Run number	Independent variable			protease activity (U/mL)	
	KH_2_PO_4_ concentration (%) X_1_	MgSO_4_ concentration (%) X_2_	pH X_3_	Observed	Predicted
1	0.07 (0)	0.01 (0)	7 (2)	1.56	3.90
2	0.105 (1)	0.005 (-1)	5.5 (-1)	2.63	3.07
3	0.07 (0)	0.01 (0)	6 (0)	4.37	4.26
4	0.07 (0)	0.01 (0)	6 (0)	4.37	4.26
5	0.035 (-1)	0.005 (-1)	5.5 (-1)	3.25	3.21
6	0.07 (0)	0 (-2)	6 (0)	3.81	3.67
7	0.07 (0)	0.02 (2)	6 (0)	4.08	3.82
8	0.07 (0)	0.01 (0)	5 (-2)	2.80	2.59
9	0.14 (2)	0.01 (0)	6 (0)	4.01	3.62
10	0.105 (1)	0.005 (-1)	6.5 (1)	3.80	3.89
11	0.105 (1)	0.015 (1)	5.5 (-1)	3.76	3.98
12	0.07 (0)	0.01 (0)	6 (0)	4.37	4.26
13	0.07 (0)	0.01 (0)	6 (0)	4.37	4.26
14	0.105 (1)	0.015 (1)	6.5 (1)	3.08	3.53
15	0.035 (-1)	0.015 (1)	5.5 (-1)	3.42	3.72
16	0.035 (-1)	0.005 (-1)	6.5 (1)	4.79	4.97
17	0.035 (-1)	0.015 (1)	6.5 (1)	4.26	4.22
18	0 (-2)	0.01 (0)	6 (0)	4.45	4.45
19	0.07 (0)	0.01 (0)	6 (0)	4.37	4.26

**Table 7:** Analysis of central composite design

Source	Sum of Squares	df	Mean square	F-value	*p*-value
Model	10.76	9	1.2	11.72	0.0006
X_1_	0.6918	1	0.6918	6.79	0.0285
X_2_	0.0235	1	0.0235	0.2302	0.6428
X_3_	1.7	1	1.7	16.66	0.0028
X_1_ × _2_	0.0774	1	0.0774	0.759	0.4063
X_1_ × _3_	0.447	1	0.447	4.38	0.0658
X_2_ × _3_	0.8062	1	0.8062	7.91	0.0203
X_1_²	0.0764	1	0.0764	0.7495	0.4091
X_2_²	0.3872	1	0.3872	3.8	0.0831
X_3_²	7.91	1	7.91	77.61	< 0.0001
**Summary of the model**					
R^2^	0.9214				
Adjusted R^2^	0.8428				
Std. Dev.	0.3193				

### Protease enzyme was characterized and showed potent proteolytic activity

**Fig. 3:**
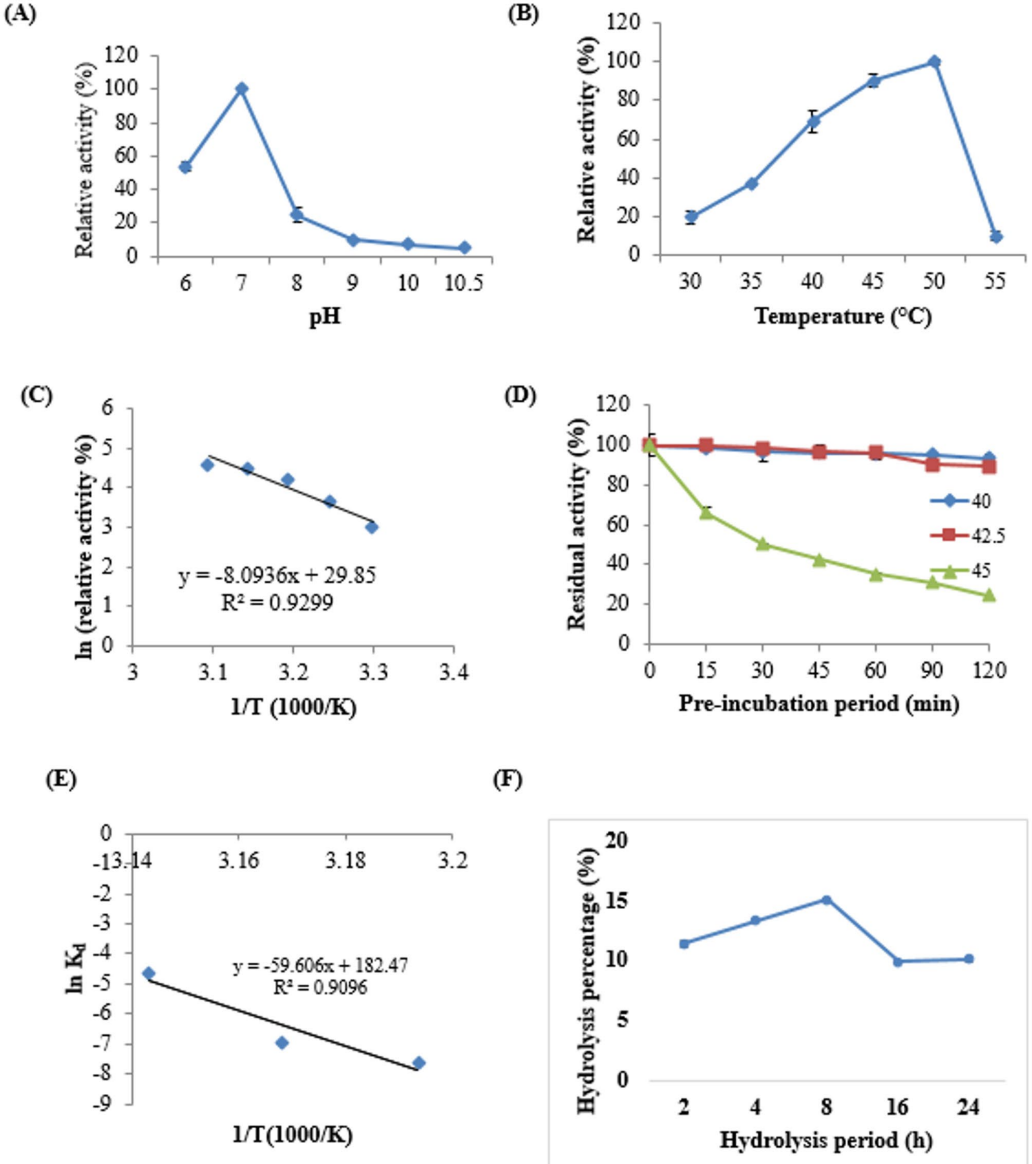
Characterization of the produced protease, (**A**) pH activity, (**B**) Temperature activity, (**C**) Arrhenius plot for thermal activation, (**D**) Temperature stability, (**E**) Arrhenius plot for thermal deactivation of the enzyme, (**F**): Enzymatic hydrolysis of FGW using the produced protease

Ethanol precipitation of the enzyme was examined, revealing that the 70% precipitated fraction had the highest specific activity (12.2 U/mg protein), with an 8-fold purification and a recovery percentage of 19.6%. The proteolytic activity of the precipitated fraction was evaluated at various pH levels (6-10.5), with pH 7 being identified as optimal for enzymatic activity (Fig. 3a), where the enzyme was completely stable. The thermal impact on proteolytic activity indicated an optimum activity at 50 °C (Fig. 3b) with an activation energy (Ea) value of 67.289 ± 4.956 KJ mol^− 1^, based on the Arrhenius plot (Fig. 3c). The thermal stability study (Fig. 3d) demonstrated the enzyme’s stability at moderate temperatures, with a half-life of 1386 min at 40 °C, which significantly decreased to 69 min at 45 °C (Table 8). Additionally, the estimated D-value was 4605 min at 40 °C, which decreased with increasing temperature, indicating rapid loss of enzymatic activity at higher temperatures. Based on the Arrhenius plot (Fig. 3e), the deactivation energy (Ed) was estimated to be 495.5 KJ mol^− 1^ for temperatures ranging from 40 to 45 °C. The applicability of the produced protease in hydrolyzing various protein substrates was examined, showing collagen and keratin hydrolyzing activity of 0.32 U/mg protein and albumin hydrolyzing activity of 0.79 U/mg protein, without any detected gelatinase activity.

**Table 8:** Thermo-kinetic parameters of the produced protease

Temperature (^ο^C)	K_d_ (min^− 1^)	T_1/2_ (min)	D-value (min)
40	0.0005	1386.3	4605.2
42.5	0.001	693.1	2302.6
45	0.01	69.3	230.3

### Production of soluble peptides from FGW

#### Enzymatic hydrolysis

By the application of the produced protease in the hydrolysis of FGW for different hydrolysis periods, the results (Fig. 3f) indicated the increase in the hydrolysis percentage by increasing the time, reaching its highest value (15%) after 8 h. For the isolation of soluble peptides, the large protein molecules were initially removed by boiling the produced hydrolysate followed by centrifugation then the clear supernatant was subjected to air drying.

#### Micro-structural analysis of FGW

**Fig. 4:**
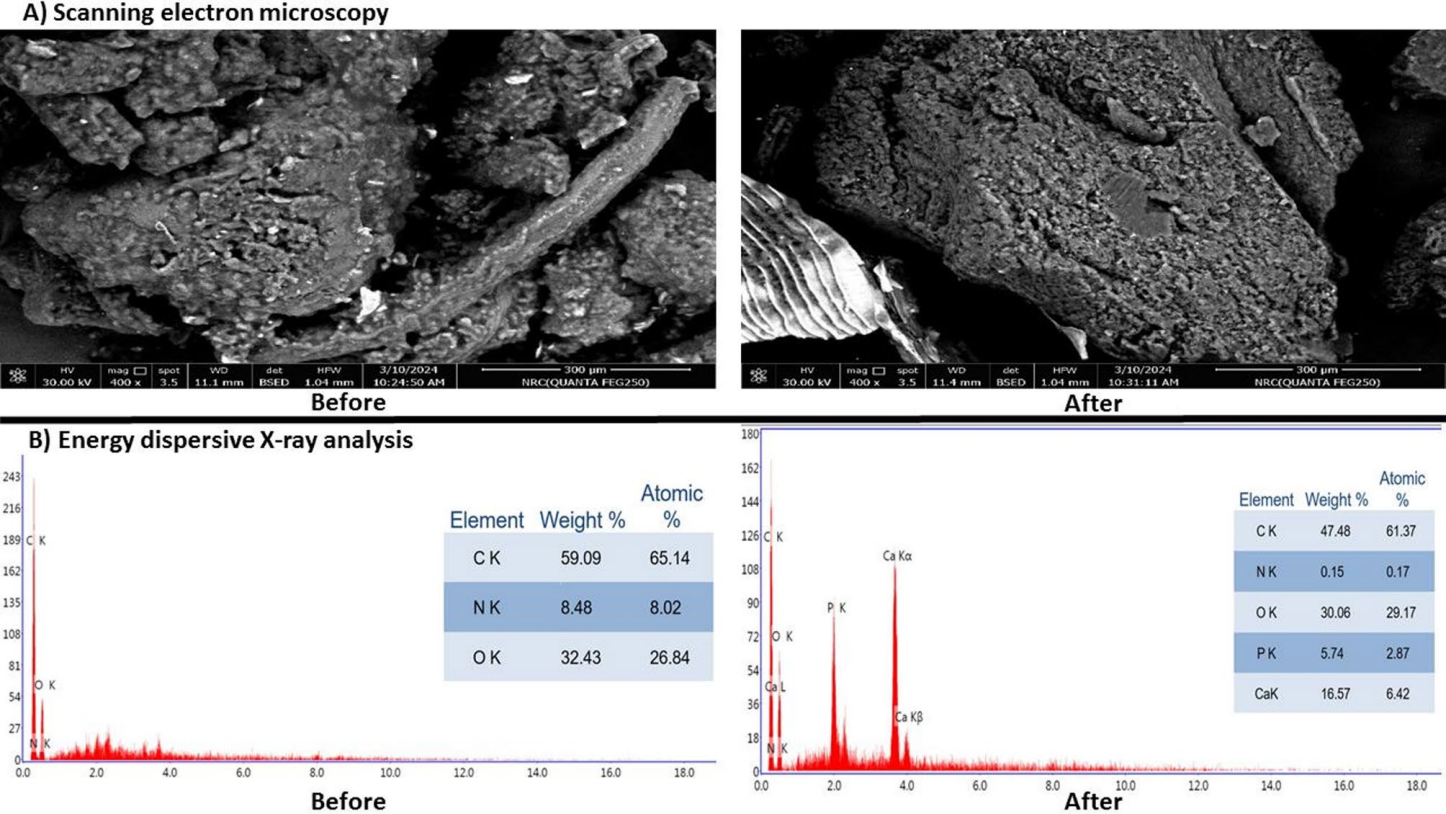
Analysis of FGW using (**A**) scanning electron microscope and (**B**) energy dispersive X-ray before and after enzymatic hydrolysis

The surface morphology of the prepared FGW after being hydrolyzed with the produced protease was examined under scanning electron microscope in compare to the un-hydrolyzed sample and the results shown in Fig. (4a) indicated no clear difference. Moreover, the elemental constituents were inspected via Energy dispersive X-ray analysis and the results indicated a significant reduction in the carbon and the nitrogen weight% in the hydrolyzed sample in compare to the un-hydrolyzed one (Fig. 4b).

#### HPLC analysis determined the amino acid composition of the produced soluble peptide sample

**Fig. 5:**
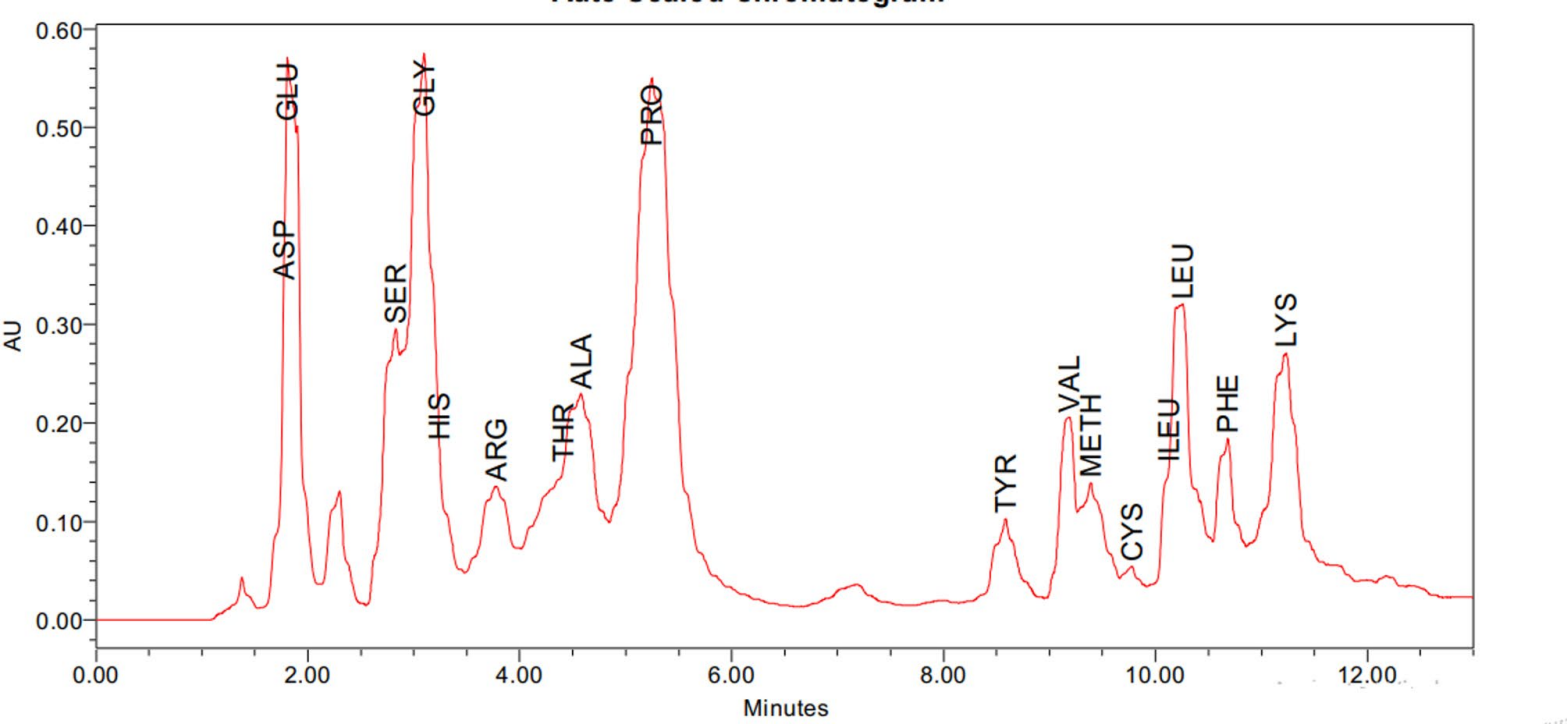
HPLC analysis of peptide sample

The structural features of soluble peptides including their length as well as their amino acid composition have significant impact on their biological activities. In the current study, the amino acid composition of the dried sample was estimated by PICO-TAG HPLC method and the results estimated the detection of 17 amino acids with various amounts (Table 9) and Figure (5). The most abundant amino acids were glutamic acid, and aspartic acid with amount equal to 230.64 and 187.03 mg/g respectively.

**Table 9:** Amino acid composition of the produced soluble peptides

Amino acid	FGW soluble peptide (mg/g)
Aspartic acid	187.03
Glutamic acid	230.64
Serine	41.26
Glycine	53.85
Histidine	38.56
Arginine	60.01
Threonine	47.51
Alanine	27.75
Proline	31.63
Tyrosine	22.36
Valine	40.72
Methionine	21.40
Cysteine	5.59
Isoleucine	46.44
Leucine	41.27
Phenylalanine	31.62
Lysine	25.60
Tryptophan not determined	

#### Proteomic analysis identified peptides with potential antifungal activity

We employed MALDI-TOF mass spectrometry to identify soluble peptides produced from the hydrolysis of FGW by a protease enzyme.

The analysis revealed the production of 138 short peptides, as shown in Table 10. Using the AMPfun predictive tool, we identified 39 peptides with potential antifungal activity (Antifungal score ≥ 0.6). AMPfun predictive tool is described as a computational tool that predicts antimicrobial activity in peptides based on their amino acid sequence and structural properties. The most abundant peptides with predicted antifungal activity were AGFAGDDAPR, VEWTDAER, AVFPSIVGRPR, FAGDDAPR, and GPIGPPGPR, with antifungal scores of 0.691, 0.61, 0.6, 0.729, and 0.641, respectively. On the other hand, the peptides with the highest potential antifungal activity were AVFPSIVGR, FAGDDAPR, LAADDFR, VNYCYTK, and AGFAGDDAPR with antifungal score of 0.77, 0.73, 0.72, 0.72 and 0.7, respectively. The antifungal score measures the likelihood of a peptide having antifungal activity, with scores ranging from 0 to 1. A score of 1 indicates the highest likelihood of antifungal activity, while a score of 0 indicates no likelihood. The cutoff value for predicting antifungal activity is 0.6.

**Table 10:** The soluble peptides produced from the hydrolysis of FGW by the produced protease enzyme

Peptide sequence	Molecular weight	Intensity percentage	Anti-fungal score
LLIVYPWAQR	1257.7	6.52	0.50
SAITSLWGK	961.5	2.85	0.56
IVLSTLGEK	958.6	2.73	0.52
AGFAGDDAPR	975.4	2.34	0.70
VEWTDAER	1004.5	2.08	0.61
AVFPSIVGRPR	1197.7	1.89	0.60
FAGDDAPR	847.4	1.78	0.73
RPEGVPQVLILITGGR	1704.0	1.69	0.37
GPIGPPGPR	862.5	1.68	0.64
IDVGEIGPQALTR	1367.7	1.54	0.25
AVFPSIVGR	944.5	1.44	0.77
ALELPEDVIK	1125.6	1.40	0.54
GIVGLPGQR	911.5	1.37	0.62
AFAGILTDADITAALAACQAADSFK	2553.2	1.29	0.36
GTYDDYVEGLR	1286.6	1.20	0.52
GATGPTGLR	828.4	1.19	0.66
DIDDLELTLAK	1244.7	1.19	0.42
SFDSIDEPASALK	1378.7	1.19	0.55
ARPTDLVFIIDSSR	1588.9	1.15	0.27
SAEIGGEALGR	1058.5	1.13	0.51
AGEPGAAGLR	913.5	1.07	0.66
LLIVYPWAQR	1314.7	1.05	0.50
LAADDFR	806.4	1.00	0.72
FLAVVVSALGR	1130.7	0.97	0.65
IQLVEEELDR	1242.6	0.97	0.64
TGPVGMPGAR	957.5	0.94	0.58
IEELEEEIEAER	1487.7	0.90	0.51
EAFGLFDR	953.5	0.89	0.59
VGVVNYASR	963.5	0.88	0.61
SNIATDIEALR	1201.6	0.87	0.35
QLGVLTIAIGTR	1223.7	0.85	0.50
DSYVGDEAQSK	1197.5	0.83	0.45
HDCDLLR	927.4	0.83	0.63
SGFIEEDELK	1165.6	0.81	0.59
VIEGLNVGPSATR	1311.7	0.80	0.56
GYTGLDGR	837.4	0.78	0.62
VTVVVITDGR	1057.6	0.78	0.50
DLTDYLMK	1013.5	0.78	0.64
GFSGLDGAK	850.4	0.78	0.68
SEIQTALEEAEGTLEHEEAK	2213.0	0.76	0.35
DAGIEIFAIGVGR	1316.7	0.76	0.45
AALTPIVESIR	1168.7	0.70	0.42
QVLILITGGR	1051.6	0.70	0.49
GIPGPIGPPGPR	1145.6	0.69	0.59
DGDVGAPGPSGPA	1111.5	0.69	0.54
TITLEVEPSDTIENVK	1786.9	0.68	0.22
GFPGLPGPSGEPGK	1327.6	0.67	0.62
SVRPSEFEQVK	1304.7	0.67	0.54
LQPVISEYYAK	1309.7	0.66	0.52
TLSDYNIQK	1080.5	0.66	0.61
SVRPENFELVK	1316.7	0.65	0.50
EALVSQLTR	1015.6	0.65	0.62
GAAGLVSPR	826.5	0.65	0.61
LGPAGPPGAR	907.5	0.65	0.60
LASYLDK	808.4	0.64	0.70
VVVITDGR	857.5	0.63	0.55
VIQCSDLGLK	1131.6	0.62	0.48
VAPEEHPTLLT	1205.6	0.62	0.46
DDDETTALVCDNGSGLVK	1950.8	0.62	0.32
IGVDEFAALVK	1160.6	0.61	0.52
DNVFTASAGSR	1123.5	0.60	0.46
AGFEDYVEGLR	1254.6	0.59	0.48
GADPEDVILSAFK	1360.7	0.58	0.44
LFLQNFSASAR	1252.7	0.57	0.54
QIIDIDECR	1143.5	0.57	0.43
GGPGIAGPTGPR	1051.5	0.56	0.59
VAIIVTDGRPQDNVK	1623.9	0.56	0.31
ALGQNPTNK	941.5	0.55	0.49
AALEQTER	916.5	0.55	0.62
QVETLSVDDGKDR	1443.7	0.54	0.50
ADIAESQVNK	1073.5	0.54	0.43
EAFTIIDQNR	1205.6	0.53	0.50
YLCSCPR	954.4	0.52	0.70
EFLEELLTTQCDR	1652.8	0.51	0.39
LQGVPQILILLTGGR	1577.0	0.51	0.50
SAVDVYLTQAK	1193.6	0.51	0.42
FASFIDK	826.4	0.51	0.69
GYSFVTTAER	1129.5	0.51	0.50
RGPTGEIGATGLAGAR	1482.8	0.50	0.49
TLCCATVGR	1036.5	0.49	0.46
ILGNPSADDLANKR	1482.8	0.48	0.50
LLIVYPWA	973.6	0.46	0.43
VGIVFTDGR	962.5	0.46	0.54
TLEDQLSELK	1174.6	0.46	0.55
AIASLWGK	844.5	0.45	0.70
VTIDTPTIDR	1129.6	0.45	0.48
TEIADLNR	930.5	0.44	0.55
NLQQEISDLTEQIGETGK	2002.0	0.44	0.30
GFPGADGGAGGK	1005.5	0.44	0.65
GEPGNPGPAGPA	1051.5	0.43	0.62
DGVNGIPGPIGPPGPR	1531.8	0.43	0.43
IVIEACTPLCPDR	1542.8	0.42	0.36
LLDQTTLTK	1031.6	0.42	0.49
VLDPAATGTIK	1084.6	0.41	0.43
GLPGSPGSSGPPGK	1241.6	0.41	0.55
EVLSAYLGK	978.5	0.41	0.55
AEDLTALR	887.5	0.41	0.57
VIADLEASYK	1107.6	0.41	0.46
VSLELFGCDH	1175.5	0.41	0.47
AAEIGGEALGR	1042.5	0.40	0.54
MLVAYPQTK	1065.6	0.39	0.48
LNFDAFLPMLK	1323.7	0.39	0.53
DLLDPVISDR	1141.6	0.39	0.67
GVTGSPGSPGPDGK	1243.6	0.39	0.54
GPAGASGPAGPR	993.5	0.38	0.65
VDALQDEINFLR	1431.7	0.37	0.56
GPTGEIGATGLAGAR	1326.7	0.37	0.49
VEGPDVDINLPK	1294.7	0.37	0.29
QLGVLTIAIGTR	1240.8	0.37	0.50
VALEQTER	944.5	0.37	0.56
SQDYIGEAAK	1080.5	0.36	0.53
QVHPDTGISSK	1150.6	0.36	0.54
LNFDAFLPMLK	1307.7	0.36	0.53
GNPGAAGSAGPQGPIGPR	1575.8	0.36	0.43
VPTPNVSVVDLTVR	1494.8	0.35	0.33
KVEAALQALTK	1170.7	0.35	0.50
LESLTDEINFLR	1448.8	0.35	0.40
TVMGGLENAVK	1133.6	0.35	0.64
IDVGEIGPQALAR	1337.7	0.33	0.37
GSPGDRGEPGPAG	1152.5	0.33	0.44
EGPAGPSGQDGR	1126.5	0.33	0.50
KLEDECSELKK	1377.7	0.33	0.61
GAAGLVSPR	883.5	0.32	0.61
GAGFAGDDAPR	1032.5	0.32	0.65
VPAVPESLLK	1051.6	0.31	0.62
CIVNIEGGK	988.5	0.31	0.55
ESTLHLVLR	1066.6	0.30	0.50
AQYEDIANR	1078.5	0.29	0.63
IQLELNQVK	1083.6	0.29	0.52
DDDNLLTYLCR	1396.6	0.29	0.48
WINQIIDK	1028.6	0.28	0.64
SAITSLWGK	1018.5	0.27	0.56
LLTDAQAEAK	1058.6	0.26	0.57
VQLELNQVK	1069.6	0.24	0.48
VNYCYTK	946.4	0.24	0.72
FDLSSFCK	1002.4	0.24	0.56
DFEISQLLSK	1178.6	0.23	0.51

### The produced peptides are effective against *C. auris*

**Fig. 6:**
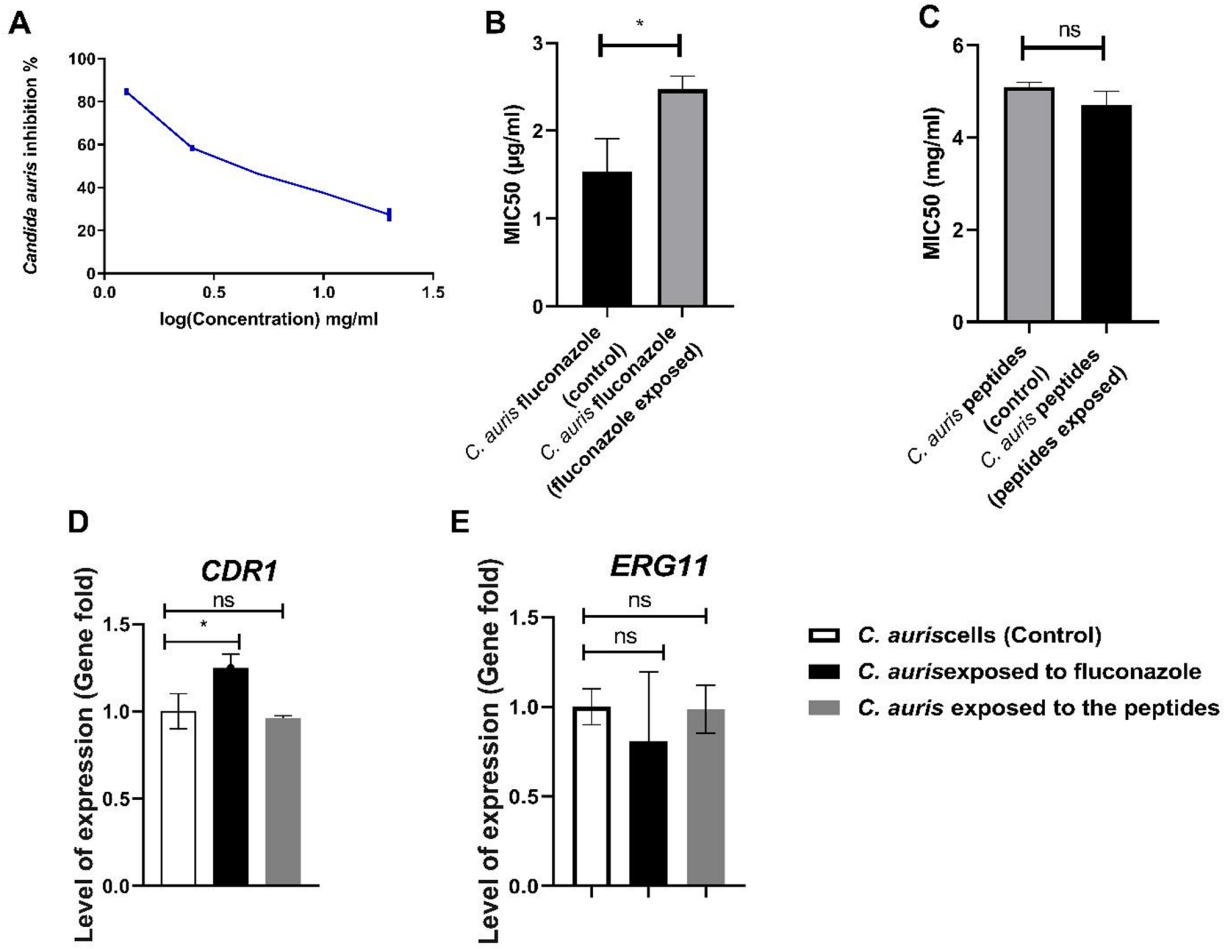
*C. auris* acquired resistance to fluconazole unlike the produced peptides. (**A**) Peptides MIC_50_ against *C. auris*, (**B**) Fluconazole MIC_50_ following repetitive exposure to fluconazole, (**C**) Peptides MIC_50_ following repetitive exposure to peptides, (**D**) Gene expression level of *CDR1* for *C. auris* either exposed to fluconazole or peptides, (**E**) Gene expression level of *ERG11* for *C. auris* either exposed to fluconazole or peptides. One-Way ANOVA was used to statistically analyze the obtained data, where *P* < 0.05, *P < 0.01, **P < 0.001, ***P < 0.0001 represent the significant levels. The data display the mean ± SD of three replicates

We exposed *C. auris* to various concentration of the produced peptides and our data showed that the produced peptides inhibited *C. auris* growth with MIC_50_ 5.1 ± 0.08 mg/ml as illustrated in Figure (6a).

### *C. auris* cannot develop resistance to produced peptides unlike fluconazole

In this experiment, we subjected *C. auris* to five exposures of both the produced peptides and fluconazole at sub-MIC levels. Our results demonstrated that the fluconazole MIC_50_ for *C. auris* significantly (*P* = 002365) increased from 1.53 ± 0.38 µg/ml to 2.47 ± 0.15 µg/ml. In contrast, exposure to the produced peptides did not alter the pathogen’s sensitivity, with the MIC_50_ remaining at 4.7 ± 0.3 mg/ml compared to 5.1 ± 0.1 mg/ml in untreated cells (see Fig. 6b and c). Additionally, gene expression analysis revealed that the *CDR1* gene was significantly overexpressed (1.24 ± 0.07) in cells exposed to fluconazole, while no significant change in expression was observed in cells exposed to the produced peptides (0.96 ± 0.013). Moreover, the expression level of the *ERG11* gene remained unchanged in cells exposed to either fluconazole (0.8 ± 0.38) or the produced peptides (0.98 ± 0.13), as illustrated in Fig. 6d and e and Additional file 1.

### Antioxidant activity

The produced peptides were found to have antioxidant activity of 50.04 ± 0.05 mmole TE/g.

### The produced peptides showed potent anti-inflammatory activity

Nitric oxide (NO) plays a significant role in the inflammatory process. We investigated the effect of the produced peptides to inhibit the release of NO from macrophages following exposure to LPS. Our data indicated the efficiency of the produced peptides to inhibit NO with IC_50_ equal to 55.8 µg/ml.

### In-vitro wound healing assay

**Fig. 7:**
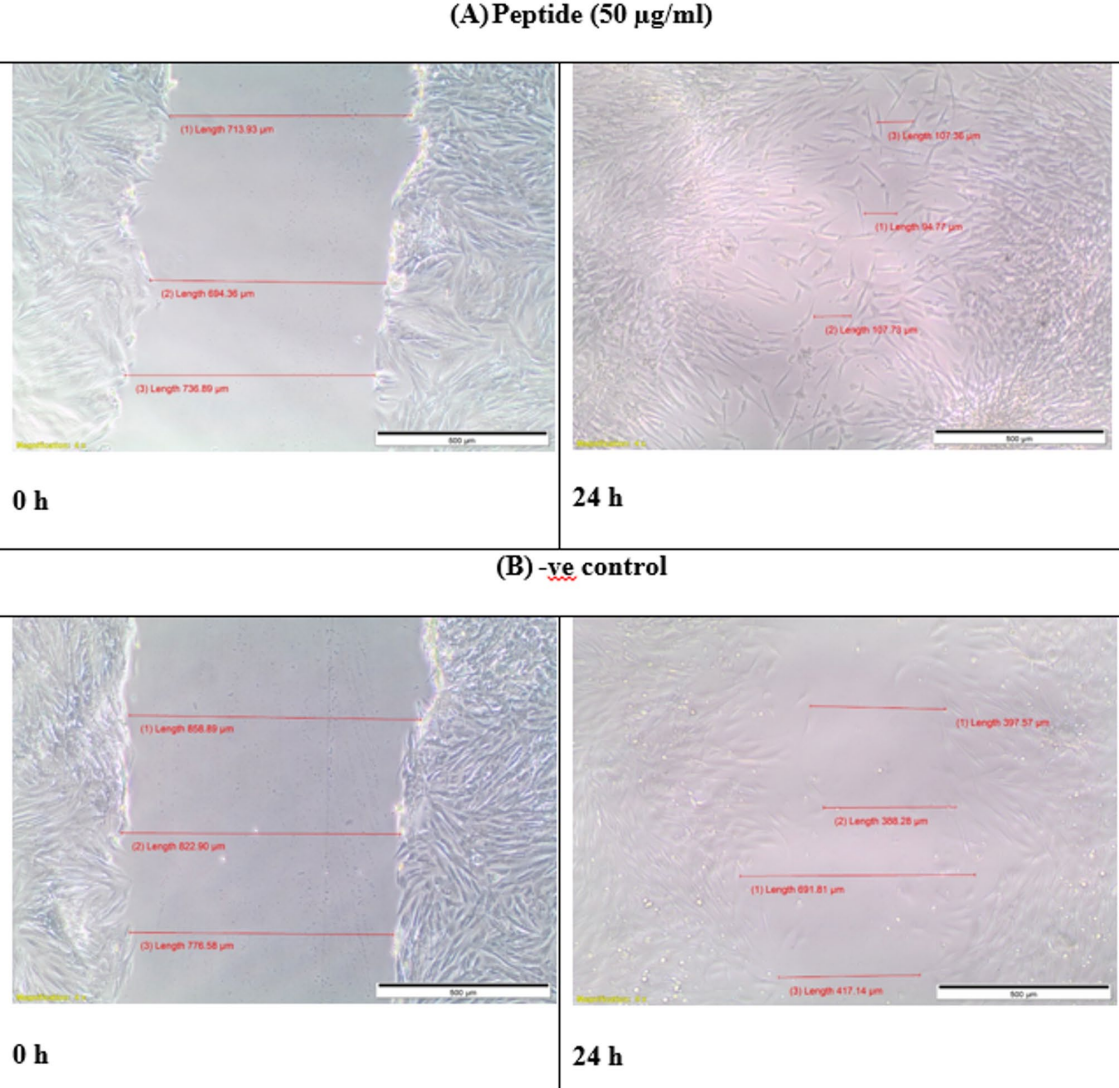
The migration of BJ-1 cells, (**A**) cells treated with peptides, (**B**) control

We have evaluated the ability of the peptides to act as wound healing agent by measuring the migration of BJ-1 cells. The image shown in Fig. (7), indicated that the wound closure percentage was 85.56% for the treated cells while that of the negative control (without addition of peptides) was 39.1% only.

## Discussion

This study introduces a new way to fight *C. auris* acquired resistance. It uses protease enzymes from *Serratia liquefaciens* NRC1 to produce antimicrobial peptides from FGW. The strain was chosen for its ability to break down casein, which is a common method for finding protease-producing strains needed to create bioactive peptides. Following screening, the strain was identified using MALDI-TOF MS and 16 S rRNA sequencing. The sequence was submitted to NCBI for public access and future research. *Serratia* species are opportunistic pathogens found in air, water, soil, and plants. They produce valuable products like enzymes, biosurfactants, and bioactive compounds useful in agriculture, food, medicine, and cosmetics [45, 46]. Specifically, *Serratia liquefaciens* isolated in the current study, has been recognized for producing various enzymes, including protease [47], lipase [48], and polyurethanase [48]. To investigate the potential of FGW as a substrate for protease enzyme production, we analyzed its protein content. Our findings corroborated those of Ramkumar et al., who confirmed that FGW is rich in protein [25]. Using biogenic materials like FGW for enzyme production has gained attention. This cost-effective method reduces enzyme production costs, making it suitable for industrial use [49–51]. Fish wastes can provide the nutritional requirements for the growth of microorganisms and production of enzymes, making them an attractive sole carbon and nitrogen source for protease production. For instance, Tran et al., used fish waste to produce protease enzyme by *Streptomyces speibonae* [52]. Similarly, Fahmy and his colleagues produced alkaline protease using fish waste as substrate [53]. Statistical optimization by applying Plackett-Burman design followed by Central composite design were employed to enhance protease production. The Plackett-Burman design allows for the rapid identification of the most significant factors affecting protease production from a large number of variables. This initial screening helps to focus resources on the most influential parameters, saving time and reducing experimental costs [40]. Once the key factors are identified, the Central Composite Design is employed to optimize these factors in detail. This method helps understand how variables interact and find the best conditions. It improves protease production efficiently [54]. Following statistical optimization, the production of protease enzyme was approximately four times higher than the protease activity before optimization. In addition, it was higher than the protease enzyme (0.22 U/mL) reported for the fermentation of 1% fish by-products of *Scarus collana*, using *Halobacillus sp. HAL1* [53], or the protease enzyme (0.64 U/mL) reported for the fermentation of 1% tuna head waste, by *Streptomyces speibonae TKU048* [52]. The observed stability of the produced enzyme at neural pH and 40-50^o^C, is common for protease enzyme produced from genus *Serratia.* For instance, protease enzyme produced by *Serratia marcescens* A3 through the fermentation of municipal solid wastes was found to be active at neutral pH and 40-50^o^C [55]. Similar observation was indicated by Wajahat et al., [56]. We observed that the produced enzyme can hydrolyze collagen and keratin as well. Proteases that can hydrolyze collagen and keratin are significant in various industrial and biomedical applications due to their ability to break down these tough and fibrous proteins. It can be used in the tanning process to soften hides and skins by breaking down collagen fibers [57]. Further, it can be **e**mployed in the wool industry to clean and process wool by degrading keratin, enhancing wool’s softness and quality [58]. Additionally, it can be utilized in meat processing to tenderize meat by breaking down collagen, enhancing texture and flavor [59].

The protease was used to create bioactive short peptides from FGW. These peptides are made up of amino acids, which can be positively charged, negatively charged, polar, or nonpolar. Notably, the analysis of soluble peptides produced in the current study showed that they consist of a mixture of amino acids with different charges and polarity. This diversity can expand the application of the produced peptides. For instance, positively charged amino acids like arginine, lysine, and histidine can make peptides antimicrobial. These amino acids help peptides attract and bind to negatively charged microbial membranes, such as phospholipids and lipopolysaccharides [60]. In contrast, biofilms often have extracellular polymeric substances with various charges. Negatively charged peptides, such as those containing glutamic acid and aspartic acid, might disrupt biofilm integrity by interacting with positively charged components. This interaction can aid in biofilm disassembly and increase the susceptibility of bacteria to other antimicrobial agents [61]. Further, MALDI-TOF/MS analysis followed by the application of the AMPfun predictive tool showed the presence of several peptides with antifungal activity. Among these, the peptide sequence AVFPSIVGR (alanine-valine-phenylalanine-proline-serine-isoleucine-valine-glycine-arginine) had the highest antifungal score. The presence of arginine at the C-terminus provides a positive charge, which is essential for interacting with the negatively charged microbial membranes. This interaction is crucial for the initial binding of AMPs to the surface of microbes [62]. Further, valine, phenylalanine, and isoleucine are hydrophobic amino acids that can interact with the lipid components of microbial membranes. This interaction is important for the insertion of the peptide into the membrane, leading to membrane disruption [63]. Additionally, proline can induce kinks, and glycine provides flexibility. These structural features can influence the peptide’s ability to adopt conformations that enhance its interaction with microbial membranes [64]. Given all this merits, the produced peptides were active against *C. auris*.

The AMPfun predictive tool, while useful for screening potential antimicrobial peptides based on their sequence, has limitations. The tool’s reliability depends on the quality of its training datasets and computational algorithms, which might not capture all biological complexities. Although AMPfun provided promising results, it is essential to validate computational predictions through experimental assays. In our study, the AMPfun predictions were experimentally validated through in vitro testing, confirming the antifungal activity of the peptides. Future studies should focus on further improving the reliability of such computational tools and incorporate experimental validation to ensure more accurate predictions of peptide efficacy and their mechanisms of action.

While *C. auris* is known for its rapid development of antifungal resistance, we found that the produced peptides did not induce resistance, even after multiple exposures. It was observed that repeated exposure to antifungal agents can lead to the development of resistance in *C. auris* [21]. *C. auris* quickly develops resistance, making treatment difficult. In the UAE, a *C. auris* case was misidentified and became resistant to antifungal drugs, leading to treatment failure and the patient’s death [18]. While several mechanisms have been proposed to contribute to acquired resistance in *C. auris*, the overexpression of efflux pump genes (e.g., *CDR1*) and antifungal target genes (e.g., *ERG11*) remains the primary identified mechanism the pathogen to either actively pump out antifungal drugs or modify drug targets, rendering conventional antifungals ineffective. This ability to quickly adapt presents a significant challenge in the clinical management of *C. auris* infections [21, 65]. We can infer from the obtained data in the present study that *C. auris* failed to acquire resistance to the peptides, unlike fluconazole, even after multiple exposures. Fluconazole specifically targets the ergosterol biosynthesis pathway, which can be bypassed or altered by overexpression of the *ERG11* gene or increased activity of efflux pumps like *CDR1*. However, our results showed that neither of these genes was overexpressed in *C. auris* following exposure to the peptides. The key difference between conventional antifungals and the peptides lies in the mode of action. Conventional antifungals like fluconazole typically target a single pathway (e.g., ergosterol synthesis), providing *C. auris* with an opportunity to adapt and develop resistance through gene mutations or overexpression. In contrast, AMPs often have multi-targeted mechanisms, attacking various components of the microbial cell simultaneously. AMPs primarily disrupt microbial membranes, causing leakage of cellular contents, which leads to cell death. This membrane disruption is not dependent on a specific biochemical pathway, making it more difficult for the pathogen to develop targeted resistance mechanisms [66]. Additionally, utilizing advanced diagnostic tools to rapidly and accurately identify *C. auris* and its resistance profile, along with regularly performing susceptibility testing, can guide effective treatment decisions [15]. We next questioned if the produced peptides exhibited additional biological activities. In this context, our data revealed that these peptides possess antioxidant, anti-inflammatory, and wound-healing properties. The peptides show antioxidant activity by neutralizing free radicals, reducing oxidative stress, and protecting cells from damage. Derived from proteins, these peptides have specific amino acid sequences enabling them to scavenge reactive oxygen species (ROS), donate hydrogen atoms or electrons, and chelate metal ions that drive oxidative reactions [67, 68]. HPLC analysis identified amino acids contributing to the antioxidant, anti-inflammatory and wound healing effects. For example, methionine contains a sulfur atom that acts as an antioxidant by donating electrons [69]. Cysteine, with its thiol (-SH) group, effectively scavenges free radicals by donating hydrogen atoms [70]. Finally, glutamic acid has carboxyl groups that contribute to antioxidant activities [71]. From the other hand, certain amino acids play significant roles in the anti-inflammatory properties of short peptides. These amino acids contribute to reducing inflammation through various mechanisms, such as modulating immune responses, scavenging free radicals, and inhibiting pro-inflammatory signaling pathways [72]. For instance, arginine is involved in the production of nitric oxide (NO), which can have anti-inflammatory effects by modulating immune cell function and vascular tone [73]. Histidine can influence immune system modulation and help regulate inflammation [74]. Glycine has anti-inflammatory properties through its role in synthesizing anti-inflammatory molecules and modulating immune responses [75]. Finally, aspartic acid can enhance the secretion of interleukin-1β (IL-1β) in M1 macrophages [76]. Similarly, amino acids in short peptides play a crucial role in wound healing by supporting various processes involved in tissue repair and regeneration. Arginine plays a vital role in collagen synthesis and the production of nitric oxide. It promotes the proliferation of fibroblasts—cells that are key to wound repair—and stimulates the production of growth factors [77]. Further, proline is a primary component of collagen, providing essential structural support to tissues. It is crucial for the formation and stabilization of collagen, which is fundamental for effective wound healing [78]. Glutamine supports cell proliferation crucial for wound healing [79]. Finally, histidine contribute to the wound healing by controlling the corticosterone and PI3K/Akt phosphorylation [80].

In considering the clinical translation of these findings, several factors need to be addressed, including the safety, stability, and scalability of these peptides for therapeutic use. While AMPs have shown great promise as therapeutic agents due to their broad-spectrum activity and low likelihood of inducing resistance, ensuring their safety is a crucial concern. Previous studies have indicated that some AMPs exhibit selective cytotoxicity toward cancer cells, without affecting normal mammalian cells [81]. However, other studies have reported potential toxicity to mammalian cells at higher concentrations [82]. Therefore, further in vivo toxicity testing is essential to determine the therapeutic window where the peptides are effective against *C. auris* but safe for human tissues.

Additionally, maintaining peptide stability in physiological environments is critical for clinical applications. AMPs are susceptible to degradation by proteases in the bloodstream or gastrointestinal tract, which can limit their therapeutic efficacy [83]. To overcome this challenge, modifications such as peptide cyclization or the incorporation of non-natural amino acids may be employed to enhance stability while retaining bioactivity. Scalability is another significant consideration for clinical translation. Producing peptides in large quantities at a reasonable cost, especially for those with complex sequences, can be challenging. However, advances in peptide synthesis techniques, including recombinant production in microbial systems and solid-phase synthesis, have improved the feasibility of large-scale production [84]. The use of sustainable substrates like FGW, as demonstrated in this study, offers an eco-friendly and cost-effective alternative that could significantly reduce production costs, making these peptides more accessible for therapeutic development.

Despite these challenges, the potential for clinical translation is promising. The U.S. Food and Drug Administration (FDA) has already approved around 100 peptides for various therapeutic applications, demonstrating the growing acceptance and feasibility of peptide-based therapies [85]. This regulatory precedent raises optimism that the data obtained in this study could contribute to the development of a novel peptide-based treatment for *C. auris*, further reinforcing the clinical potential of AMPs in combating drug-resistant infections.

Future research should focus on further characterization of the bioactive peptides, including detailed studies on their mechanisms of action and potential synergistic effects with existing antifungal agents. Further, while we faced challenges in synthesizing the most active peptide identified in this study, we recognize the importance of this step in validating our findings in the future. This will help to further investigate the therapeutic potential of the identified antimicrobial peptides and assess their efficacy against *C. auris* in clinical settings. Also,

expanding the scope of peptide applications to other pathogens and assessing their efficacy in clinical settings could provide valuable insights. Additionally, exploring the genetic and biochemical pathways involved in peptide synthesis and activity may yield new strategies for enhancing peptide production and functionality. Investigating the long-term stability and safety of these peptides in various formulations could pave the way for their incorporation into therapeutic and industrial products.

## Conclusion

This study demonstrates the potential of using FGW as a sustainable substrate for producing AMPs via protease enzymes derived from *Serratia liquefaciens* NRC1. The peptides generated showed promising antifungal activity against *C. auris*. Notably, *C. auris* did not develop resistance to the peptides, unlike conventional antifungal agents such as fluconazole, highlighting the therapeutic promise of these AMPs. In addition to their antifungal efficacy, these peptides possess other beneficial biological activities, including antioxidant, anti-inflammatory, and wound-healing properties. This broad spectrum of bioactivity positions the peptides as potential candidates for commercial development, not only as antifungal treatments but also as wound-healing agents and other biomedical applications. Moreover, their versatility could extend to industries such as cosmetics and food preservation, where antimicrobial properties are highly valuable. Future investigations should focus on elucidating the molecular mechanisms by which *Serratia* metabolites, including antimicrobial peptides and enzymes, exert their antifungal effects. This may include transcriptomic, proteomic, and metabolomic studies to uncover the cellular pathways and targets affected in pathogenic fungi. Further, in vivo studies are required to validate the therapeutic potential of these peptides.

## Electronic supplementary material

Below is the link to the electronic supplementary material.

Supplementary Material 1: Raw Ct values. Description of data: Raw Ct values for qPCR analysis

## Data Availability

Sequencing data obtained from 16 S rRNA sequencing analysis was submitted to GenBank under the name Serratia liquefaciens NRC1 and acquired an accession number PP038128.

## References

[1] Coppola D, Lauritano C, Palma Esposito F, Riccio G, Rizzo C, de Pascale D. Fish waste: from problem to valuable resource. Mar Drugs. 2021;19(2). 10.3390/md19020116 PMC7923225 33669858

[2] Ben Rebah F, Miled N. Fish processing wastes for microbial enzyme production: a review. 3 Biotech. 2013;3(4):255–65. 28324586 10.1007/s13205-012-0099-8 PMC3723863

[3] Ghaly A, Ramakrishnan V, Brooks M, Budge S. Dave djaa, review. oAc. Fish processing wastes as a potential source of proteins. Microb Biochem Technol. 2013;5(4):107–29.

[4] Venugopal V. Enzymes from seafood processing waste and their applications in seafood processing. Adv Food Nutr Res. 2016;78:47–69. 27452165 10.1016/bs.afnr.2016.06.004

[5] Dhole NP, Phuge S, Dar MA, Pandit RS. Screening of chitin degrading bacteria from the gut of Asian common Toad Duttaphrynus melanostictus: implication for valorization of chitin rich seafood waste. Environ Technol Innov. 2022;28:102929.

[6] Dhole NP, Dar MA, Pandit RS. Recent advances in the bioprospection and applications of chitinolytic bacteria for valorization of waste chitin. Arch Microbiol. 2021;203(5):1953–69. 33710379 10.1007/s00203-021-02234-5

[7] Akbarian M, Khani A, Eghbalpour S, Uversky VN. Bioactive peptides: synthesis, sources, applications, and proposed mechanisms of action. Int J Mol Sci. 2022;23(3). 10.3390/ijms23031445 PMC8836030 35163367

[8] Krell T, Matilla MA. Antimicrobial resistance: progress and challenges in antibiotic discovery and anti-infective therapy. Microb Biotechnol. 2022;15(1):70–8. 34610194 10.1111/1751-7915.13945 PMC8719825

[9] Xuan J, Feng W, Wang J, Wang R, Zhang B, Bo L, et al. Antimicrobial peptides for combating drug-resistant bacterial infections. Drug Resist Updates: Reviews Commentaries Antimicrob Anticancer Chemother. 2023;68:100954. 10.1016/j.drup.2023.100954 36905712

[10] Huan Y, Kong Q, Mou H, Yi H. Antimicrobial peptides: classification, design, application and research progress in multiple fields. Front Microbiol. 2020;11:582779. 33178164 10.3389/fmicb.2020.582779 PMC7596191

[11] Omardien S, Drijfhout JW, Vaz FM, Wenzel M, Hamoen LW, Zaat SAJ, et al. Bactericidal activity of amphipathic cationic antimicrobial peptides involves altering the membrane fluidity when interacting with the phospholipid bilayer. Biochim Et Biophys Acta Biomembr. 2018;1860(11):2404–15. 10.1016/j.bbamem.2018.06.004 29902419

[12] He SW, Zhang J, Li NQ, Zhou S, Yue B, Zhang M. A TFPI-1 peptide that induces degradation of bacterial nucleic acids, and inhibits bacterial and viral infection in half-smooth tongue sole, Cynoglossus semilaevis. Fish Shellfish Immunol. 2017;60:466–73. 27840169 10.1016/j.fsi.2016.11.029

[13] Satoh K, Makimura K, Hasumi Y, Nishiyama Y, Uchida K, Yamaguchi H. Candida auris sp. nov., a novel ascomycetous yeast isolated from the external ear canal of an inpatient in a Japanese hospital. Microbiol Immunol. 2009;53(1):41–4. 19161556 10.1111/j.1348-0421.2008.00083.x

[14] Pallotta F, Viale P, Barchiesi F. Candida auris: the new fungal threat. Infez Med. 2023;31(3):323–8. 37701386 10.53854/liim-3103-6 PMC10495051

[15] Fayed B, Lazreg IK, AlHumaidi RB, Qasem MA, Alajmy BMGN, Bojbarah FM, et al. Intra-clade heterogeneity in Candida auris: risk of management. Curr Microbiol. 2023;80(9):295. 37486431 10.1007/s00284-023-03416-8

[16] Fayed B, Shakartalla SB, Sabbah H, Dalle H, Tannira M, Senok A, et al. Transcriptome analysis of human dermal cells infected with Candida auris identified unique pathogenesis/defensive mechanisms particularly ferroptosis. Mycopathologia. 2024;189(4):65. 38990436 10.1007/s11046-024-00868-9

[17] de Jong AW, Hagen F, Attack. Defend and persist: how the fungal pathogen Candida auris was able to emerge globally in healthcare environments. Mycopathologia. 2019;184(3):353–65. 31209693 10.1007/s11046-019-00351-w

[18] Alatoom A, Sartawi M, Lawlor K, AbdelWareth L, Thomsen J, Nusair A, et al. Persistent candidemia despite appropriate fungal therapy: first case of Candida auris from the United Arab Emirates. Int J Infect Dis. 2018;70:36–7. 29452247 10.1016/j.ijid.2018.02.005

[19] Zhu Y, O’Brien B, Leach L, Clarke A, Bates M, Adams E et al. Laboratory analysis of an outbreak of Candida auris in New York from 2016 to 2018: impact and lessons learned. J Clin Microbiol. 2020;58(4). 10.1128/JCM.01503-19 PMC7098748 31852764

[20] Hamdy R, Fayed B, Hamoda AM, Rawas-Qalaji M, Haider M, Soliman SS. Essential oil-based design and development of novel anti-Candida Azoles formulation. J Molecules. 2020;25(6):1463. 10.3390/molecules25061463 PMC7146627 32213931

[21] Fayed B, Jayakumar MN, Soliman SS. Caspofungin-resistance in Candida auris is cell wall-dependent phenotype and potential prevention by zinc oxide nanoparticles. Med Mycol J. 2021;59(12):1243–56. 10.1093/mmy/myab059 34612496

[22] Rybak JM, Muñoz JF, Barker KS, Parker JE, Esquivel BD, Berkow EL et al. Mutations in TAC1B: a novel genetic determinant of clinical fluconazole resistance in Candida auris. mBio. 2020;11(3). 10.1128/mBio.00365-20 PMC7218281 32398311

[23] Reda A, Fayed B. The synthesis of calcium doped zinc oxide ceramic nanoparticles via sol–gel effective against the emerging multidrug-resistant Candida auris. ACSJ. 2023;59(5):1315–23.

[24] Kumar S, Stecher G, Li M, Knyaz C, Tamura K. MEGA X: molecular evolutionary genetics analysis across computing platforms. Mol Biol Evol. 2018;35(6):1547–9. 29722887 10.1093/molbev/msy096 PMC5967553

[25] Ramkumar A, Sivakumar N, Gujarathi AM, Victor R. Production of thermotolerant, detergent stable alkaline protease using the gut waste of Sardinella longiceps as a substrate: optimization and characterization. Sci Rep. 2018;8(1):12442. 30127443 10.1038/s41598-018-30155-9 PMC6102305

[26] Kirk PL. Kjeldahl method for total nitrogen. Anal Chem. 1950;22(2):354–8.

[27] Folch J, Lees M, Sloane Stanley GH. A simple method for the isolation and purification of total lipides from animal tissues. J Biol Chem. 1957;226(1):497–509. 13428781

[28] Dubois M, Gilles K, Hamilton JK, Rebers PA, Smith F. A colorimetric method for the determination of sugars. Nature. 1951;168(4265):167. 14875032 10.1038/168167a0

[29] Pojić M, Kravić S, Stojanović Z, Nollet L, Toldra F. Analytical methods for determination of moisture and ash in foodstuffs. Handbook of food analysis. Volume 1. CRC; 2015. pp. 275–96.

[30] Horikoshi K. Production of alkaline enzymes by alkalophilic microorganisms: part i. alkaline protease produced by Bacillus 221. Agri Bio Chem. 1971;35(9):1407–14.

[31] Boughachiche F, Zerizer H, Tiouche A, Gramez A, Ait Kaki A, Rachedi K. Statistical optimization of culture medium for neutral protease production by Streptomyces sp. using sardine viscera. Iran J Fish Sci. 2024;23(4):519–36.

[32] Tennalli GB, Hungund BS, Jain AM. Response surface methodology mediated optimization of serine protease by Salinicola tamaricis BGN2 isolated from the West Coast of India and its application in culturing of MCF7 cell line. J Appl Pharm Sci. 2024;14(3):119–35.

[33] Plackett RL, Burman JP. The design of optimum multifactorial experiments. Biometrika. 1946;33(4):305–25.

[34] Box G, Behnken D. Simplex-sum designs: a class of second order rotatable designs derivable from those of first order. Ann Math Stat. 1960;31(4):838–64.

[35] Lowry OH, Rosebrough NJ, Farr AL, Randall RJ. Protein measurement with the Folin phenol reagent. J Biol Chem. 1951;193(1):265–75. 14907713

[36] White JA, Hart RJ, Fry JC. An evaluation of the waters pico-tag system for the amino-acid analysis of food materials. J Automat Chem. 1986;8(4):170–7. 18925132 10.1155/S1463924686000330 PMC2547673

[37] Saadeldin IM, Swelum AA, Elsafadi M, Mahmood A, Osama A, Shikshaky H, et al. Thermotolerance and plasticity of camel somatic cells exposed to acute and chronic heat stress. J Adv Res. 2020;22:105–18. 31969994 10.1016/j.jare.2019.11.009 PMC6965514

[38] Sameh M, Khalaf HM, Anwar AM, Osama A, Ahmed EA, Mahgoub S, et al. Integrated multiomics analysis to infer COVID-19 biological insights. Sci Rep. 2023;13(1):1802. 36720931 10.1038/s41598-023-28816-5 PMC9888750

[39] Chung CR, Kuo TR, Wu LC, Lee TY, Horng JT. Characterization and identification of antimicrobial peptides with different functional activities. Brief Bioinform. 20. 10.1093/bib/bbz043 31155657

[40] Ismail SA, Fayed B, Abdelhameed RM, Hassan AA. Chitinase-functionalized UiO-66 framework nanoparticles active against multidrug-resistant Candida auris. BMC Microbiol. 2024;24(1):269. 39030474 10.1186/s12866-024-03414-1 PMC11264975

[41] Livak KJ, Schmittgen TD. Analysis of relative gene expression data using real-time quantitative PCR and the 2(-Delta Delta C(T)) method. Methods. 2001;25(4):402–8. 11846609 10.1006/meth.2001.1262

[42] Brand-Williams W, Cuvelier M-E, Berset C. Use of a free radical method to evaluate antioxidant activity. LWT. 1995;28(1):25–30.

[43] Yoon WJ, Kim SS, Oh TH, Lee NH, Hyun CG. Abies Koreana essential oil inhibits drug-resistant skin pathogen growth and LPS-induced inflammatory effects of murine macrophage. Lipids. 2009;44(5):471–6. 19350303 10.1007/s11745-009-3297-3

[44] Tam JC, Lau KM, Liu CL, To MH, Kwok HF, Lai KK, et al. The in vivo and in vitro diabetic wound healing effects of a 2-herb formula and its mechanisms of action. J Ethnopharmacol. 2011;134(3):831–8. 21291991 10.1016/j.jep.2011.01.032

[45] Mander L, Liu H-W. Comprehensive natural products II: chemistry and biology. Elsevier; 2010.

[46] Bodade RG. Secondary metabolites from Serratia sp. and their applications. Bacterial Secondary Metabolites: Elsevier; 2024. pp. 259–75.

[47] Machado SG, Heyndrickx M, De Block J, Devreese B, Vandenberghe I, Vanetti MC, et al. Identification and characterization of a heat-resistant protease from Serratia liquefaciens isolated from Brazilian cold raw milk. Int J Food Microbiol. 2016;222:65–71. 26874224 10.1016/j.ijfoodmicro.2016.01.014

[48] Salgado CA, Silva JG, Almeida FA, Vanetti MCD. Biodegradation of polyurethanes by Serratia liquefaciens L135 and its polyurethanase: in silico and in vitro analyses. Environ Pollut. 2023;333:122016. 37339733 10.1016/j.envpol.2023.122016

[49] Emran MA, Ismail SA, Abdel-Fattah AM. Valorization of feather via the microbial production of multi-applicable keratinolytic enzyme. Biocatal Agric Biotechnol. 2020;27:101674.

[50] Hassan AA, Ismail SA. Production of antifungal N-acetyl-β-glucosaminidase chitinolytic enzyme using shrimp byproducts. Biocatal Agric Biotechnol. 2021;34:102027.

[51] Ismail SA, Nour SA, Hassan AA. Valorization of corn cobs for Xylanase production by Aspergillus flavus AW1 and its application in the production of antioxidant oligosaccharides and removal of food stain. Biocatal Agric Biotechnol. 2022;41:102311.

[52] Tran TN, Doan CT, Nguyen VB, Nguyen AD, Wang S-L. Conversion of fishery waste to proteases by Streptomyces speibonae and their application in antioxidant preparation. Fishes. 2022;7(3):140.

[53] Fahmy NM, El-Deeb B. Optimization, partial purification, and characterization of a novel high molecular weight alkaline protease produced by Halobacillus sp. HAL1 using fish wastes as a substrate. J Genet Eng Biotechnol. 2023;21(1):48. 37121925 10.1186/s43141-023-00509-6 PMC10149429

[54] Gopalakrishnan D, Jain A. A statistical and downstream approach for the improvement of protease production from Bacillus toyonensis VKB5 isolated from Actinidia deliciosa. JMBFS. 2021;11(1).

[55] Iqbal A, Hakim A, Hossain MS, Rahman MR, Islam K, Azim MF, et al. Partial purification and characterization of serine protease produced through fermentation of organic municipal solid wastes by Serratia marcescens A3 and pseudomonas putida A2. J Genet Eng Biotechnol. 2018;16(1):29–37. 30647701 10.1016/j.jgeb.2017.10.011 PMC6296650

[56] Wajahat SS, Azim MK, Saleem F. Characterization of metalloprotease from Serratia marcescens. PJBMB. 2022;55(2):128–37.

[57] Lasoń-Rydel M, Sieczyńska K, Gendaszewska D, Ławińska K, Olejnik TP. Use of enzymatic processes in the tanning of leather materials. Autex Res. 2024;24(1):20230012.

[58] Gunes G, Akkoyun O, Demir T, Bozaci E, Demir A, Hames EE. Microbial keratinase production and application to improve the properties of wool fabrics. Int J Text Sci. 2018;7(2):43–7.

[59] Miller A, Strange E, Whiting R. Improved tenderness of restructured beef steaks by a microbial collagenase derived from Vibrio B-30. Food Sci. 1989;54(4):855–7.

[60] Lei J, Sun L, Huang S, Zhu C, Li P, He J, et al. The antimicrobial peptides and their potential clinical applications. Am J Translational Res. 2019;11(7):3919–31. PMC6684887 31396309

[61] Warraich AA, Mohammed AR, Perrie Y, Hussain M, Gibson H, Rahman A. Evaluation of anti-biofilm activity of acidic amino acids and synergy with ciprofloxacin on Staphylococcus aureus biofilms. Sci Rep. 2020;10(1):9021. 32488138 10.1038/s41598-020-66082-x PMC7265346

[62] Chan DI, Prenner EJ, Vogel HJ. Tryptophan- and arginine-rich antimicrobial peptides: structures and mechanisms of action. Biochim Biophys Acta. 2006;1758(9):1184–202. 16756942 10.1016/j.bbamem.2006.04.006

[63] Schmidt NW, Wong GC. Antimicrobial peptides and induced membrane curvature: geometry, coordination chemistry, and molecular engineering. Curr Opin Solid State Mater Sci. 2013;17(4):151–63. 24778573 10.1016/j.cossms.2013.09.004 PMC4000235

[64] Tuerkova A, Kabelka I, Králová T, Sukeník L, Pokorná Š, Hof M et al. Effect of helical kink in antimicrobial peptides on membrane pore formation. eLife. 2020;9. 10.7554/eLife.47946 PMC7069690 32167466

[65] Bhattacharya S, Holowka T, Orner EP, Fries BC. Gene duplication associated with increased fluconazole tolerance in Candida auris cells of advanced generational age. Sci Rep. 2019;9(1):5052. 30911079 10.1038/s41598-019-41513-6 PMC6434143

[66] Kean R, Ramage G. Combined antifungal resistance and biofilm tolerance: the global threat of Candida auris. mSphere. 2019;4(4). 10.1128/mSphere.00458-19 PMC6669339 31366705

[67] López-García G, Dublan-García O, Arizmendi-Cotero D. Gómez Oliván LM. Antioxidant and antimicrobial peptides derived from food proteins. Molecules. 2022;27(4). 10.3390/molecules27041343 PMC8878547 35209132

[68] Zhang Y, Li Y, Quan Z, Xiao P, Duan JA. New insights into antioxidant peptides: an overview of efficient screening, evaluation models, molecular mechanisms, and applications. Antioxidants (Basel, Switzerland). 2024;13(2). 10.3390/antiox13020203 PMC10886007 38397801

[69] Aledo JC. Methionine in proteins: the Cinderella of the proteinogenic amino acids. Protein Sci. 2019;28(10):1785–96. 31359525 10.1002/pro.3698 PMC6739822

[70] Park CM, Weerasinghe L, Day JJ, Fukuto JM, Xian M. Persulfides: current knowledge and challenges in chemistry and chemical biology. Mol Biosyst. 2015;11(7):1775–85. 25969163 10.1039/c5mb00216h PMC4470748

[71] Yang Y, Wang R, Ai X, Liu D, Niu C, Li T. Significant enhancement in antioxidant and antimicrobial activity of tragacanth gum through chemical modification using amino acids. Int J Biol Macromol. 2024;257(Pt 1):128343. 38007020 10.1016/j.ijbiomac.2023.128343

[72] Buccini DF, Roriz BC, Rodrigues JM, Franco OL. Antimicrobial peptides could antagonize uncontrolled inflammation via toll-like 4 receptor. Front Bioeng Biotechnol. 2022;10:1037147. 36568291 10.3389/fbioe.2022.1037147 PMC9767961

[73] Jiang H, Xu Q, Wang X, Shi L, Yang X, Sun J, et al. Preparation of antibacterial, arginine-modified ag nanoclusters in the hydrogel used for promoting diabetic, infected wound healing. ACS Omega. 2023;8(14):12653–63. 37065086 10.1021/acsomega.2c07266 PMC10099449

[74] Moro J, Tomé D, Schmidely P, Demersay TC, Azzout-Marniche D. Histidine: a systematic review on metabolism and physiological effects in human and different animal species. Nutrients. 2020;12(5). 10.3390/nu12051414 PMC7284872 32423010

[75] Aguayo-Cerón KA, Sánchez-Muñoz F, Gutierrez-Rojas RA, Acevedo-Villavicencio LN, Flores-Zarate AV, Huang F et al. Glycine: the smallest anti-inflammatory micronutrient. Int J Mol Sci. 2023;24(14). 10.3390/ijms241411236 PMC10379184 37510995

[76] Wang H, Zheng X, Liu B, Xia Y, Xin Z, Deng B, et al. Aspartate metabolism facilitates IL-1β production in inflammatory macrophages. Front Immunol. 2021;12:753092. 34745126 10.3389/fimmu.2021.753092 PMC8567039

[77] Murakami H, Shimbo K, Inoue Y, Takino Y, Kobayashi H. Importance of amino acid composition to improve skin collagen protein synthesis rates in UV-irradiated mice. Amino Acids. 2012;42(6):2481–9. 21861170 10.1007/s00726-011-1059-z PMC3351609

[78] Karna E, Szoka L, Huynh TYL, Palka JA. Proline-dependent regulation of collagen metabolism. Cell Mol Life Sci. 2020;77(10):1911–8. 31740988 10.1007/s00018-019-03363-3 PMC7228914

[79] Malakar P, Singha D, Choudhury D, Shukla S. Glutamine regulates the cellular proliferation and cell cycle progression by modulating the mTOR mediated protein levels of β-TrCP. Cell Cycle (Georgetown Tex). 2023;22(17):1937–50. 37771151 10.1080/15384101.2023.2260166 PMC10599172

[80] Kim Y, Kim E, Kim Y. l-histidine and l-carnosine accelerate wound healing via regulation of corticosterone and PI3K/Akt phosphorylation in d-galactose-induced aging models in vitro and in vivo. Funct Food. 2019;58:227–37.

[81] Hoskin DW, Ramamoorthy A. Studies on anticancer activities of antimicrobial peptides. Biochim Biophys Acta. 2008;1778(2):357–75. 18078805 10.1016/j.bbamem.2007.11.008 PMC2238813

[82] Soundrarajan N, Park S, Le Van Chanh Q, Cho HS, Raghunathan G, Ahn B, et al. Protegrin-1 cytotoxicity towards mammalian cells positively correlates with the magnitude of conformational changes of the unfolded form upon cell interaction. Sci Rep. 2019;9(1):11569. 31399625 10.1038/s41598-019-47955-2 PMC6689069

[83] Garvey M. Antimicrobial peptides demonstrate activity against resistant bacterial pathogens. Infect Dis Rep. 2023;15(4):454–69. 37623050 10.3390/idr15040046 PMC10454446

[84] Wang L, Wang N, Zhang W, Cheng X, Yan Z, Shao G, et al. Therapeutic peptides: current applications and future directions. Signal Transduct Target Therapy. 2022;7(1):48. 10.1038/s41392-022-00904-4 PMC8844085 35165272

[85] Al Musaimi O, Exploring FDA-A, Frontiers. Insights into natural and engineered peptide analogues in the GLP-1, GIP, GHRH, CCK, ACTH, and α-MSH realms. Biomolecules. 2024;14(3). 10.3390/biom14030264 PMC10968328 38540684

